# The Flexible Fairness: Equality, Earned Entitlement, and Self-Interest

**DOI:** 10.1371/journal.pone.0073106

**Published:** 2013-09-09

**Authors:** Chunliang Feng, Yi Luo, Ruolei Gu, Lucas S. Broster, Xueyi Shen, Tengxiang Tian, Yue-Jia Luo, Frank Krueger

**Affiliations:** 1 State Key Laboratory of Cognitive Neuroscience and Learning, Beijing Normal University, Beijing, China; 2 Key Laboratory of Behavioral Science, Institute of Psychology, Chinese Academy of Sciences, Beijing, China; 3 Institute of Affective and Social Neuroscience, Shenzhen University, Shenzhen, China; 4 Sichuan Research Center of Applied Psychology, Chengdu Medical College, Chengdu, China; 5 Department of Behavioral Science, University of Kentucky College of Medicine, Lexington, Kentucky, United States of America; 6 Department of Psychology, George Mason University, Fairfax, Virginia, United States of America; 7 Department of Molecular Neuroscience, George Mason University, Fairfax, Virginia, United States of America; University of Bologna, Italy

## Abstract

The current study explored whether earned entitlement modulated the perception of fairness in three experiments. A preliminary resource earning task was added before players decided how to allocate the resource they jointly earned. Participants’ decision in allocation, their responses to equal or unequal offers, whether advantageous or disadvantageous, and subjective ratings of fairness were all assessed in the current study. Behavioral results revealed that participants proposed more generous offers and showed enhanced tolerance to disadvantageous unequal offers from others when they performed worse than their presumed “partners,” while the reverse was true in the better-performance condition. The subjective ratings also indicated the effect of earned entitlement, such that worse performance was associated with higher perceived feelings of fairness for disadvantageous unequal offers, while better performance was associated with higher feelings of fairness for advantageous unequal offers. Equal offers were considered “fair” only when earned entitlement was even between two parties. In sum, the perception of fairness is modulated by an integration of egalitarian motivation and entitlement. In addition to justice principles, participants were also motivated by self-interest, such that participants placed more weight on entitlement in the better-performance condition than in the worse-performance condition. These results imply that earned entitlement is evaluated in a self-serving way.

## Introduction

It is widely acknowledged that human behavior often deviates from the goal of maximizing self-benefit during social decision-making [1]. Substantial evidence from economics [2], evolutionary biology [3,4], and social neuroscience [5–7] has implicated other-regarding preference (i.e., fairness) in resource allocation. Speficially, individuals’ perferences for fairness have been revealed in two classical games: dictator game (DG) and ultimate game (UG). For example, in the DG, the dictator decides how to split a certain windfall between him/herself and the receiver, who can only accept the dectator’s decision [8,9]. In contrast to maximizing their own self-interest, dictators show a preference for equality and offer approximately 20% (rather than nothing) of the windfall to responders [10]. Such an egalitarian motivation appears in human beings as early as 7-8 years old such that children avoid both advantageous and disadvantageous inequality when they decide how to distribute hedonic rewards (e.g., candy) between themselves and their partners [11]. In the UG, the offer proposed by the proposer can be either accepted or rejected by the responder. The money is split as proposed if the offer is accepted, whereas both proposer and responder receive zero following rejection [12]. In contrast to the standard presumption of self-interest (i.e., that responders will accept any positive offer), the rejection rate reaches 50% when the offer is below 30% of the windfall [10]. Therefore, experimental evidence from both classical DG and UG has identified egalitarian motivation in human beings [2].

However, individuals in the classical DG/UG task always make “context-free” decisions, such that the resources for allocation are “manna from heaven” [13]. Furthermore, the fairness principle in these tasks is framed in its simplest form: equality of outcomes. Though the simplicity inherent in these classical forms of the DG/UG game was viewed favorably in earlier studies, the same simplicity has been called into question in recent years [14,15]. In particular, resource allocation in the real world is typically based not on the windfall profits, but rather on resources jointly earned. More importantly, the egalitarian motivation revealed by classical DG/UG task cannot reflect the pluralism of fairness principles in the real world [16]. Fairness principles are heterogeneous among individuals [17], and economic decisions from a single person may also reflect the trade-off between multiple fairness principles [18]. This view is supported by recent evidence that fairness principles adopted by individuals are flexible across contexts [19], such that individuals integrate both preferences for equality and contextual cues (e.g., merit, expectation) to form a context-specific fairness motivation for decision-making [20]. In short, the simplicity of classical DG/UG task reduces the ecological validity of these tasks and may lead to inconsistency between individuals’ decisions in the laboratory setting and in the real world [21,22].

To address these issues, an increasing number of studies have added a production phase before the allocation phase [23–28]. That is, the surplus allocated in the allocation phase is earned from the production phase. This procedure provides a promising way to measure distinct fairness principles by manipulating performance in the allocation phase. The performance manipulation leads to senses of asymmetry between players regarding earned entitlement to the surplus [25]. In this context, individuals show little preference for equality, but rather allocate the surplus according to that sense of entitlement [29]. That is, proposers who have earned the entitlement on the surplus will allocate very little to their opponents. Such a pinciple of earned entitlement has been well established in previous studies regarding distributive justice [17,30]. However, individuals may also take advantage of performance manipulation to increase their payoffs (i.e., self-interestes). In particular, individuals who earn higher entitlement are more prone to take the factor of entitlement into consideration during resource allocation; whereas entitlement is much less weighted when individuals are at lower levels of perceived entitlement [18,31].

Here, we replicated the modulations of earned entitlement on individuals’ decisions in resource allocation and examined whether entitlement would influence participants’ responses to unequal offers. This study aimed to extend current literature on distributive justice in the following aspects: Firstly, both DG and UG tasks were used in the current study. Previous studies predominately focused on how entitlement modulates resource distribution (i.e., dictators’ decisions in the DG task), whereas limited studies have reported effects of entitlement on individuals’ responses to unequal offers (i.e., responders’ costly punishment in the UG task). Individuals’ decisions in these two tasks are based on distinct cognitive fucntions: DG-dictators’ decisions are primarily linked to cognitive-control processes [32,33]; whereas UG-responders’ decisions are closely associated with affective responses [6,34,35] (but see 35,36. Therefore, it remains unknown whether entitlement modulates both types of decisions in a similar way. Secondly, participants in this study played multiple rounds of DG/UG (Experiments 1 and 2). In this regard, the current study aimed to develop a paradigm that could be directly combined with neuroimaging techniques, such as functional magnetic resonance imaging (fMRI). Most neuroimaging studies on fairness have been conducted with classical DG/UG tasks [1]; thus, few neuroimaging studies have investigated the modulations of entitlement on preference for equality. However, participants often play only one round in most previous behavioral studies focusing on the entitlement aspect of distributive justice, and this kind of one-round task is unsuitable for neuroimaging studies. Importantly, individuals’ decisions in the multi-round DG/UG task were confirmed with a further study consisting of one-shot version of DG/UG (Experiment 3), so that the consistency of participants’ decisions across ditinct versions of DG/UG could be directly measured. Finally, participants’ subjective ratings of fairness in each condition were collected in the current study. These subjective ratings, which can help to the interpret underlying psychological mechanism of economic decisions [34,37], have been ignored in most recent studies.

In summary, the current study investigated whether and how entitlement modulated individuals’ allocation decisions in the DG/UG and their responses to unequal offers in the UG. We added a resource-earning task before the stage of resource distribution to manipulate levels of performance. According to previous studies, participants would make their decisions based on the interaction of egalitarian motivation, earned entitlement, and self-interest (strategic motivation and self-serving bias) [38]. Egalitarian motivation entails that people will always show preference for equality, irrespective of levels of entitlement (see also 29. However, individuals may consider both earned entitlement and preference for equality rather than following one simple principle such as equal shares. In particular, participants might allocate less surplus to themselves and show more tolerance to unequal offers in the worse-performance condition than better-performance condition and vice versa, even though these decisions would lead to unequality between two players [17,30,39]. Furthermore, participants might act in a self-serving way to increase their payoff, such that earned entitlement would be emphasized to a larger extent when participants’ performance was better, compared to the condition when participants’ perormance was worse [18,31]. Accordingly, the money units that proposers allocate to themselves in the better-performance condition would be more than the money units that proposers would allocate to receipients in the worse-performance condition. In the same vein, responders would accept more advantageous unequal offers in the better-performance condition compared with the acceptance rate of disadvantageous unequal offers in the worse-performance condition. Finally, the money units participants allocated to themselves were expected to be higher in the DG compared with those allocated in the UG. This difference between UG and DG would reflect individual’s strategic motivation in the UG, given that the proposer of UG has to consider potential sanction when making offers [33,40]. In summary, the preference for equality might be modulated by both earned entitlement and self-interest.

## Experiment 1

### Methods: Experiment 1

#### Ethics Statement

This study was completely approved by the Institutional Review Board (IRB) at Beijing Normal University. Written informed consents were collected for all participants.

#### Participants

Sixty-seven college students aged 18–26 years (51 females; mean age 21.22 ± 1.84 years) participated in Experiment 1 as paid volunteers.

#### Task Procedure

Participants were asked to complete the tasks simultaneously with three other anonymous persons sitting in different rooms with the doors closed. Participants finished the tasks on a personal computer. Stimulus display and behavioral data acquisition were conducted using PsychToolbox (http://psychtoolbox.org/PTB-2/) in the Matlab environment [41,42]. Participants were told that the computer would randomly assign a “partner” for them from one of the three persons at the beginning of each round. To encourage participants to make real decisions, they were informed that the money units (MUs) accrued during the game would increase the bonus money received at the end of the experiment. In reality, in order to control for partner strategy across participants, participants were playing with a pre-programmed computer algorithm (see also 43.

Experiment 1 contained 120 rounds in total, with 105 rounds consisting of two steps: resource earning and resource distribution (see also 44. Resource earning was represented in the form of a number estimation task (NET), while resource distribution was represented in the form of the traditional ultimatum game (UG) and dictator game (DG). In the other 15 rounds, the stage of resource distribution was skipped (see below).

#### The Number Estimation Task (NET)

On each round, fixation was first presented at the center of the screen for 1000 ms. Then participants saw a screen with 100 red dots for 100 ms. The screen was divided equally into left and right halves by a black line. For each round, the number of red dots (100 in total and varied between 40 and 60 in each side) was slightly different between the left and right sides, but discriminating the difference was visually difficult (see Figure S1). Participants were instructed to judge which side had more dots, by pressing “F” with left index finger to indicate that there were more dots on the left side, and “J” with right index finger to indicate as much on the right side (for similar game settings, see [45,46]). After a delay of 800-1200 ms, the feedback of the NET was presented for 2000 ms.

There were four types of feedback in the NET: “you-right, other-right”, “you-right, other-wrong”, “you-wrong, other-right”, and “you-wrong, other-wrong” (i.e., where “you” refers to the participant and “other” refers to the presumed partner). Each participant was told that he/she and his/her partner would together get a reward of 100 MUs if either of them made the right choice in the task (“you-right, other-right”, “you-right, other-wrong”, or “you-wrong, other-right”). Then they would be directed to the step of resource distribution (i.e., the UG/DG) after 1800-2200 ms. Otherwise, for the “you-wrong, other-wrong” feedback, participants received no reward, and the UG/DG tasks would be skipped. Participants’ reaction times (RT) and accuracy (ACC) of the NET in three experiments can be obtained from supplementary information (see Figure S6, S7, S8, S9, S10 and Text S1).

The NET feedback was used to manipulate levels of performance. Participants’ performance was better than their partner in the “you-right, other-wrong” condition, but worse than their partner in the “you-wrong, other-right” condition. Unbeknownst to participants, feedback was pre-determined by the computer and was independent of participants’ real performance. Each kind of feedback was presented in 35 rounds except the “you-wrong, other-wrong” feedback, which appeared in 15 rounds.

#### The Ultimatum Game (UG) and Dictator Game (DG)

The UG is a classical paradigm for investigating fairness [12]. In the UG, one player (the proposer) receives an endowment and proposes how to split the money between him/herself and the other player (the responder), who starts with zero. The responder is free to accept or reject the offer. Each player gets the proposed share if the offer is accepted; whereas both players get nothing following a rejection. The DG is similar with the UG, except that DG responders may not reject offers, so proposers may ignore potential rejection [8,9]. However, the UG/DG task employed in the current study differed from the typical UG/DG in the sense that players are randomly awarded in the classical games, but reward used for UG/DG is earned by players in the current study.

As described above, the UG/DG task contained 105 rounds in which the NET feedback was not “you-wrong, other-wrong” (90 rounds for the UG and 15 rounds for the DG). For the participant and his/her partner in the NET, one of them would act as the UG/DG proposer, and the other as the responder. To avoid the potential effect of personal reputation, participants were told that, when playing the role of UG proposer, their offer on each round would be immediately accepted or rejected by their partner, but they would not know their partner’s decision until the end of the experiment [47]. Likewise, when playing the role of the responder, the partner would not learn the participants’ decision on each round. Participants played as UG proposers in 15 rounds, UG responders in 75 rounds, and DG proposers in 15 rounds. The sequence of these three types of rounds was pseudorandom and pre-determined.

When playing as UG/DG proposers, participants were asked to allocate the 100 MUs by pressing one of the buttons “1-9” on the keyboard (see Figure S4). For example, pressing “6” meant that participants kept 60 MUs for themselves and left 40 MUs to their partner (see Figure S2).

When playing as UG/DG responders, participants were instructed to accept or reject the offers from their “partner” by pressing the “F” or “J” buttons, respectively, or the reverse button assignments (counterbalanced across participants). In each round, one of five potential offers would be presented: “you-50, other-50”, “you-40, other-60”, “you-30, other-70”, “you-20, other-80”, or “you-10, other-50”. Like the NET feedback, the offers were actually pre-determined by the computer (rather than by other persons) in a pseudorandom sequence, with each kind of offer appearing in 15 rounds. After participants made the decision, the resulting allocation would be presented for 1000 ms (see Figure S3).

Following the formal tasks, participants were asked to rate the levels of fairness (7-point scale) of each kind of offer. At the end of the experiment, each participant was paid 30 Chinese yuan (approximately five dollars) as compensation. In addition, all participants were completely debriefed about the deception and the experiment’s motivation.

#### Statistics

Three kinds of dependent variables were entered into data analysis: (a) participants’ proposed offers in the UG/DG proposer stage, (b) participants’ acceptance rate in the responder stage, and (c) the results of the self-rating fairness. 

For all the analyses listed below, the significance level was set at 0.05 (two-tailed). Repeated measures analysis of variance (ANOVA) was used for statistical analysis [34,48], with the within-subject factors of performance (better *vs.* even *vs.* worse) and game (UG *vs.* DG) for proposed offers; and performance (better *vs.* even *vs.* worse) and offer (50:50 vs. 40:60 vs. 30:70 vs. 20:80 vs. 10:90) for acceptance rate and subjective ratings. Greenhouse–Geisser correction for ANOVA tests was used whenever appropriate. Post-hoc testing of significant effects was conducted using the Bonferroni method. Statistical analysis was performed using SPSS Statistics 16.0 (IBM, Somers, USA). Only significant effects are reported hereafter.

### Results: Experiment 1

#### Resources Allocation: Proposals

**Figure 1:**
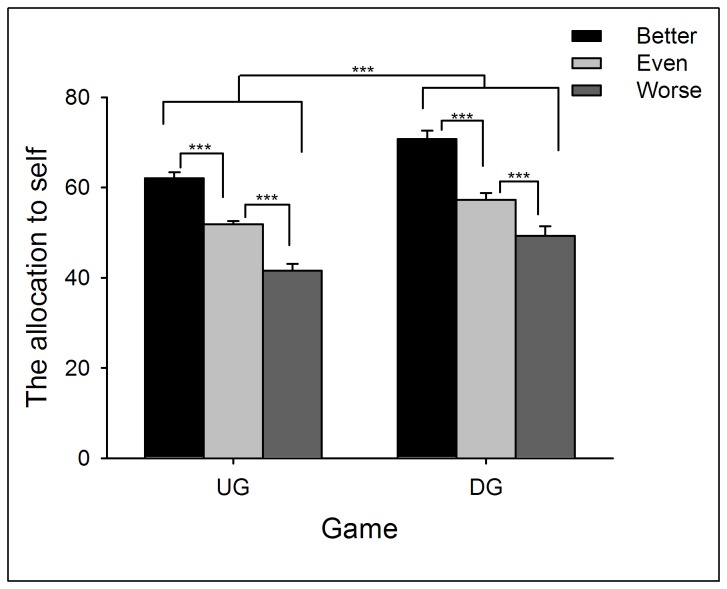
The average points participants allocated to themselves in the UG and DG in Experiment 1. The better the participants’ performance, the more they allocated to themselves. In addition, participants allocated more to themselves in DG than UG Error bars indicate 1 SE. UG: ultimate game; DG: dictator game.

Two-way repeated measures ANOVA of Performance (better *vs.* even *vs.* worse) by Game (UG *vs.* DG) yielded significant main effects of Performance (*F*(2,132) = 76.66, *p* < .0005) and Game (*F*(1,66) = 30.23, *p* < .0005). Participants’ allocation to themselves was largest in the better-performance condition (in which their performance was better than their partner), and smallest in the worse-performance condition (*p* < .05). In addition, participants allocated themselves more MUs in the DG than in the UG (*p* < .05; see Figure 1 and Table S1; see also [49]). In addition, as a DG proposer, participants’ allocation to themselves in the better-performance condition was larger than the MUs participants allocated to their partner in the worse-performance condition (*t*(66) = 6.67, *p* < .0005).

#### Responses to Offers: Acceptance Rate

Two-way repeated measures ANOVA of Performance (better *vs.* even *vs.* worse) by Offer (50:50 vs. 40:60 vs. 30:70 vs. 20:80 vs. 10:90) yielded significant main effects of Performance (*F*(2,132) = 63.01, *p* < .0005) and Offer (*F*(4,264) = 94.29, *p* < .0005) and a significant interaction between Performance and Offer (*F*(8,528) = 12.84, *p* < .0005). The acceptance rate decreased as a function of levels of performance but increased as a function of allocations to participants.

**Figure 2:**
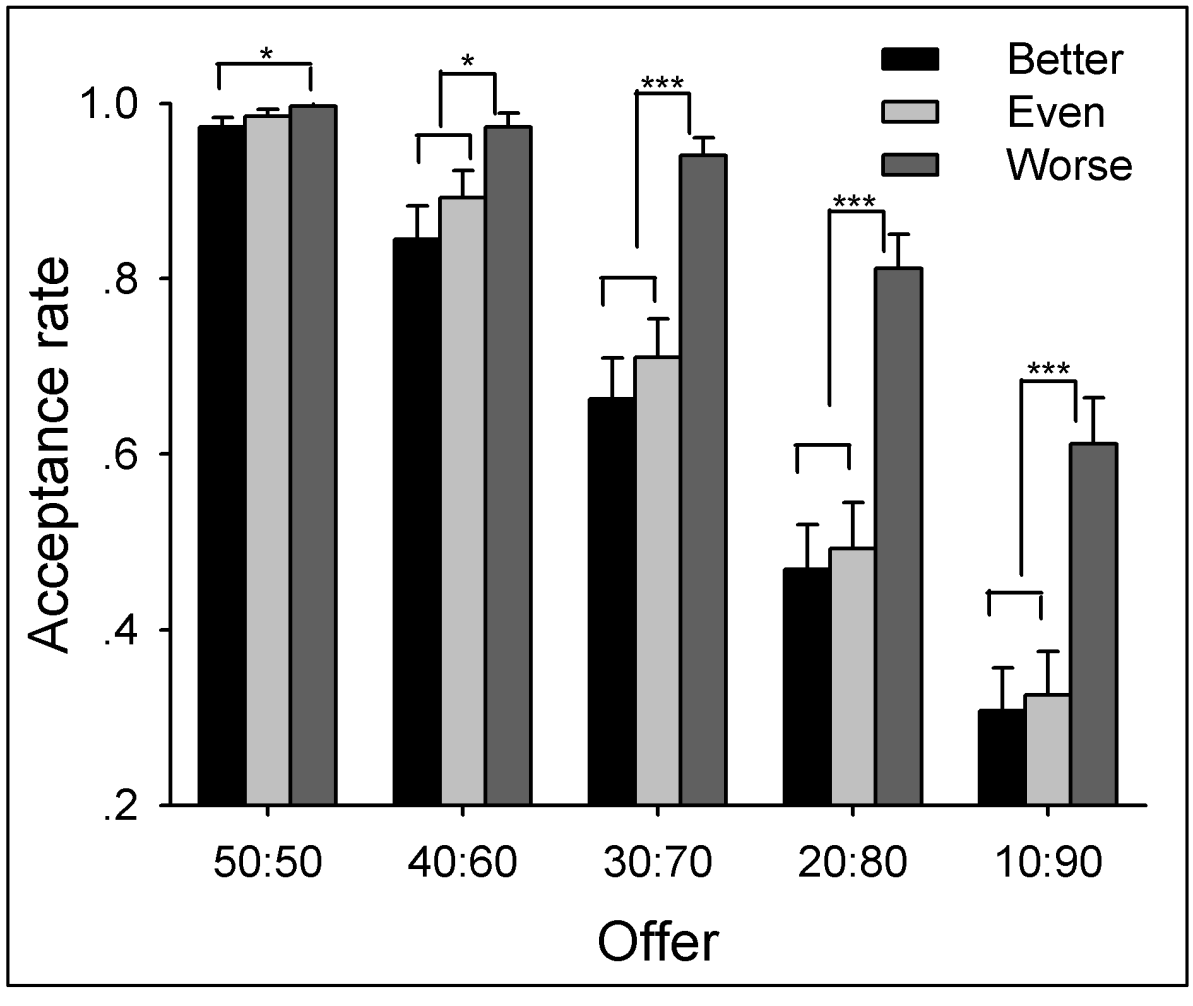
The average acceptance rate to different kinds of offers within each performance in Experiment 1. Participants showed enhanced tolerance to unequal offers when their performance was worse than their partners, such that the acceptance rate was higher in the worse-performance condition compared with the better-performance and even-performance conditions in all unequal offers. The acceptance rate did not show any difference between the even-performance condition and the better-performance condition for any offers. Error bars indicate 1 SE.

Simple-effect analysis on the interaction revealed that, regarding the factor of Performance, the acceptance rate in the worse-performance condition was higher than both the better- and even-performance conditions for all kinds of offers (*p* < .05) except equal offers (50:50), for which the worse-performance condition was higher than the better-performance condition (*p* < .05) but showed no difference with the even-performance condition (*p* > .05) (see Figure 2 and Table S2). In addition, the difference between the better- and the even-performance conditions was not significant for any kind of offer (*p* > .05).

Regarding the factor of Offer, the acceptance rate for equal offers was higher than other kinds of offers in the better- and even-performance conditions (*p* < .05) whereas no significant difference was found between the offers 50:50, 40:60, and 30:70 in the worse-performance condition (*p* > .05).

#### Fairness Ratings

**Figure 3:**
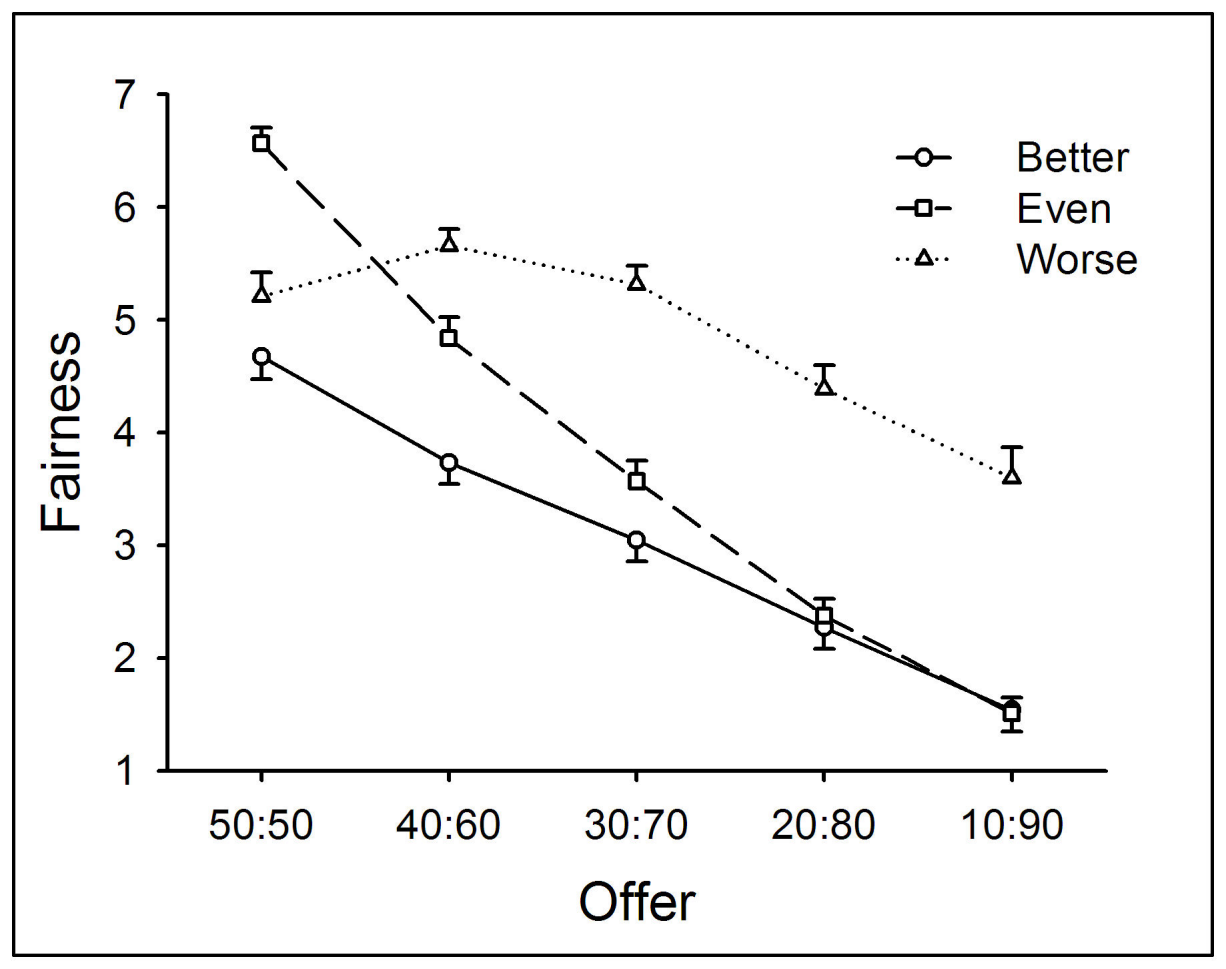
The fairness ratings in response to different kinds of offers within each performance condition in Experiment 1. The ratings of fairness decreased as offers became more unequal and as performance became better. Error bars indicate 1 SE.

Two-way repeated measures ANOVA of Performance (better *vs.* even *vs.* worse) by Offer (50:50 vs. 40:60 vs. 30:70 vs. 20:80 vs. 10:90) yielded significant main effects of Performance (*F*(2,132) = 55.58, *p* < .0005) and Offer (*F*(4,264) = 141.97, *p* < .0005) and a significant interaction between Performance and Offer (*F*(8,528) = 31.36, *p* < .0005). Feelings of fairness decreased as a function of levels of performance but increased as a function of allocations to participants. Simple-effect analysis on the interaction revealed that for equal offers (50:50), the fairness rating was higher in the even-performance condition than the other two conditions (*p* < .05); for moderately unequal offers (40:60 and 30:70), the rating was highest in the worse-performance condition, and lowest in the better-performance condition (*p* < .05); for highly unequal offers (20:80 and 10:90), the rating was higher in the worse-performance condition than the other two conditions (*p* < .05) (see Figure 3).

### Discussion: Experiment 1

When playing in the UG and DG, participants’ behavior was significantly affected by their performance during resource earning (i.e., from the NET task). If participants’ performance was worse than their partners, they offered more generous allocations when playing the role of UG/DG proposers, and showed more tolerance to unequal offers when playing the role of UG responders. In contrast, if participants’ performance was better than their partners, they were less likely to propose an equal offer (see also [17,44,50]). These results reveal that individuals agree to make resource distribution according to each party’s earned entitlement. However, the extent to which entitlement was considered during decision-making was motivated by self-interest, such that participants allocated more MUs to themselves in the better-performance condition than they allocated to their partner in the worse-performance condition. These findings indicated that individuals are more prone to take entitlement into consideration in the better-performance condition than worse-performance condition.

Similarly, proposers allocated more to themselves in the DG than in the UG. Indeed, the differences in allocation between UG and DG have been considered as an elegant index of strategic motivation, because the proposer does not face potential punishment (i.e., rejection) in the DG; thus, no strategic behavior is needed in the DG. In contrast, punishment threat in the UG is likely to induce fear of rejection and corresponding strategic motivation [40].

Regarding the subjective-rating scales, the fairness rating decreased as a function of levels of performance, indicating that entitlement modulated fairness judgments. The classical “egalitarianism motivation” is also evidenced, such that the fairness rating for the equal offer (50:50) was highest when participants’ performance was the same as their partners. Nevertheless, in the worse-performance condition, participants rated 50:50 less fair than 40:60 (see Figure 3). In conclusion, feelings of fairness reflected an integration between the classical “egalitarianism motivation” and earned entitlement.

In Experiment 1, the most generous offer in the UG task was 50:50, and unequal offers were always disadvantageous to participants. However, we noticed that the fairness rating in the better-performance condition stayed at a relatively low level even when the offer was 50:50 (see Figure 3), indicating that when participants’ performance was better, 50:50 was not “fair enough” to them. Indeed, when playing as proposers, participants tended to allocate more MUs to their partners than to themselves in the worse-performance condition (see Figure 1). Thus, we suggest that levels of performance also modulate participants’ perception of fairness to unequal offers that are advantageous to them.

Previous studies suggest that proposers avoid both advantageous and disadvantageous unequal resources allocation [11]. Likewise, responders reject unequal offers that are advantageous to themselves, indicating the egalitarianism motivation [51–54]. However, the earned entitlement was not explicitly manipulated in these studies, so it remained unclear whether people would consider unequal, advantageous offers as “fair proposals” when they performed better than their partners. In order to clarify this point, UG-responders were exposed to both advantageous and disadvantageous offers in the Experiment 2.

## Experiment 2

### Methods: Experiment 2

#### Ethics Statement

This study was completely approved by the Institutional Review Board (IRB) at Beijing Normal University. Written informed consents were collected for all participants.

#### Participants

Seventy college students aged 18–24 years (42 females; mean age 21.59 ± 2.05 years) participated in Experiment 2 as paid volunteers. None of these students participated in Experiment 1.

#### Task Procedure

In general, the task procedure was the same as Experiment 1, except that the potential offers to UG-responders included distributions more favorable than 50:50. Specifically, potential offers included: “you-90, other-10”, “you-70, other-30”, “you-50, other-50”, “you-30, other-70”, and “you-10, other-90” (see Figure S5).

### Results: Experiment 2

#### Resources Allocation: Proposals

**Figure 4:**
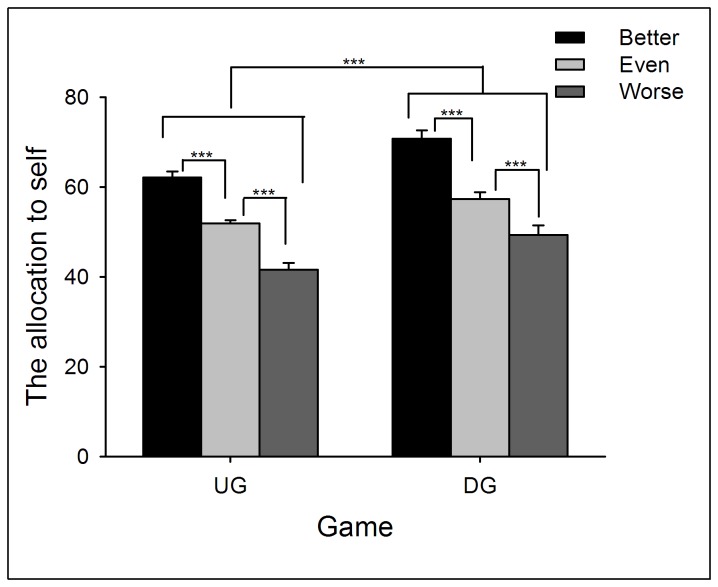
The average points participants allocated to themselves in the UG and DG in Experiment 2. The points that participants allocated to themselves increased with the performance. In addition, participants allocated more to themselves in the DG than in the UG. Error bars indicate 1 SE. UG: ultimate game; DG: dictator game.

Two-way ANOVA of Performance (better *vs.* even *vs.* worse) by Game (UG *vs.* DG) yielded significant main effects of Performance (*F*(2,138) = 134.63, *p* < .0005) and Game (*F*(1,69) = 34.43, *p* < .0005). Similar with Experiment 1, participants’ allocation to themselves was largest in the better-performance condition, and smallest in the worse-performance condition (*p* < .05). In addition, participants allocated themselves more MUs in the DG than in the UG (see Figure 4 and Table S3). In addition, as a DG proposer, participants’ allocation to themselves in the better-performance condition was larger than that to the partner in the worse-performance condition (*t*(69) = 6.08, *p* < .0005).

#### Responses to Offers: Acceptance Rate

**Figure 5:**
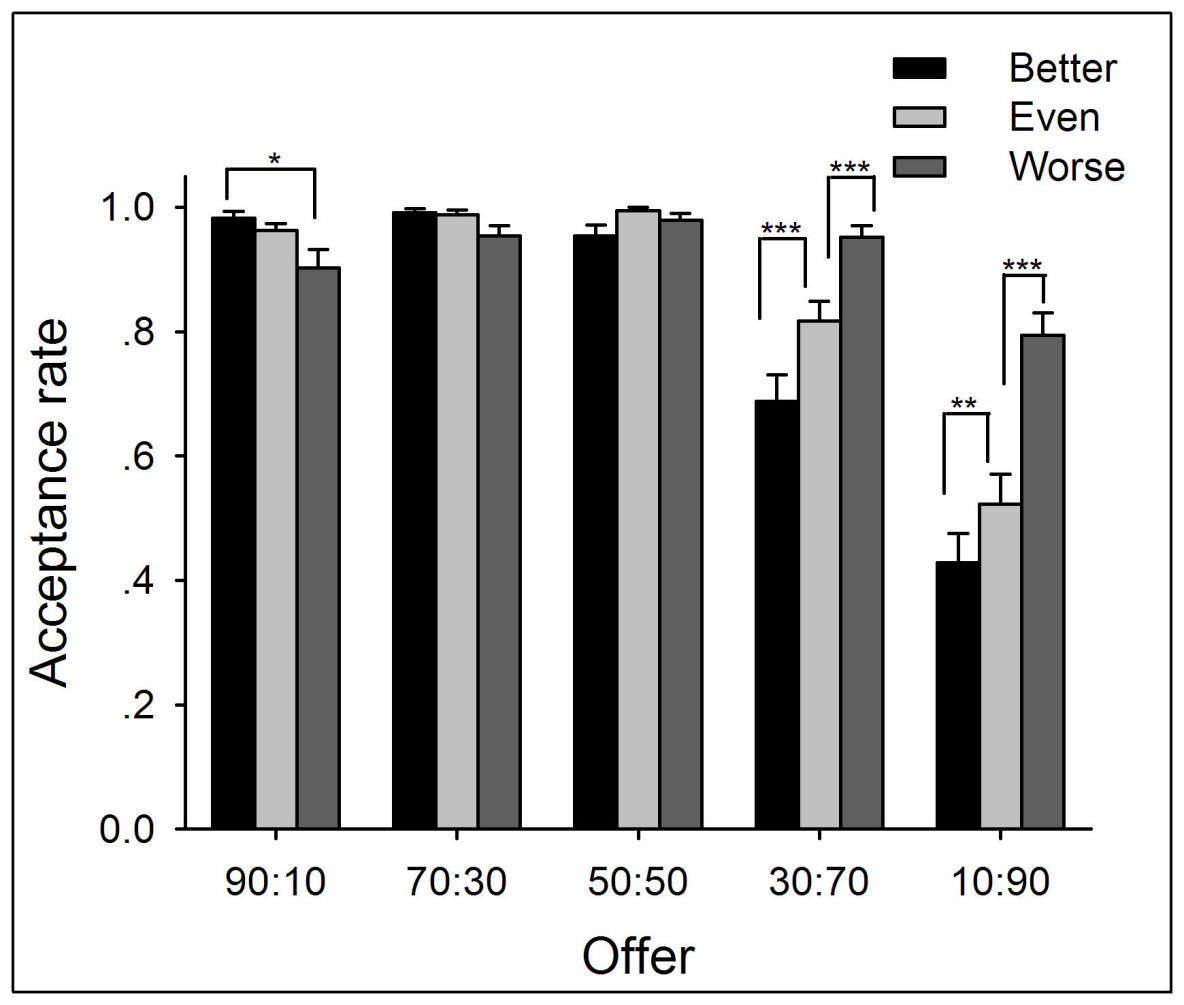
The average acceptance rates to different kinds of offers within each performance condition in Experiment 2. Earned entitlement modulated participants’ response to both advantageous and disadvantageous unequal offers, such that the acceptance rate was lowest in the worse-performance condition in response to advantageous unequal offers and highest in the worse-performance condition in response to disadvantageous unequal offers. Error bars indicate 1 SE.

Two-way repeated measures ANOVA of Performance (better *vs.* even *vs.* worse) by Offer (90:10 *vs.* 70:30 *vs.* 50:50 *vs.* 30:70 *vs.* 10:90) yielded significant main effects of Performance (*F*(2,138) = 41.25, *p* < .0005) and Offer (*F*(4,276) = 80.24, *p* < .0005) and a significant interaction between Performance and Offer (*F*(8,552) = 29.17, *p* < .0005). The acceptance rate decreased as a function of levels of performance but increased as a function of allocations to participants. Simple-effect analysis on the interaction revealed that, for 90:10 (advantageous) offers, the acceptance rate was lower in the worse-performance condition compared with the better-performance condition (*p* < .05). For disadvantageous offers (30:70 and 10:90), the acceptance rate was highest in the worse-performance condition, and lowest in the better-performance condition (*p* < .05) (see Figure 5 and Table S4).

**Figure 6:**
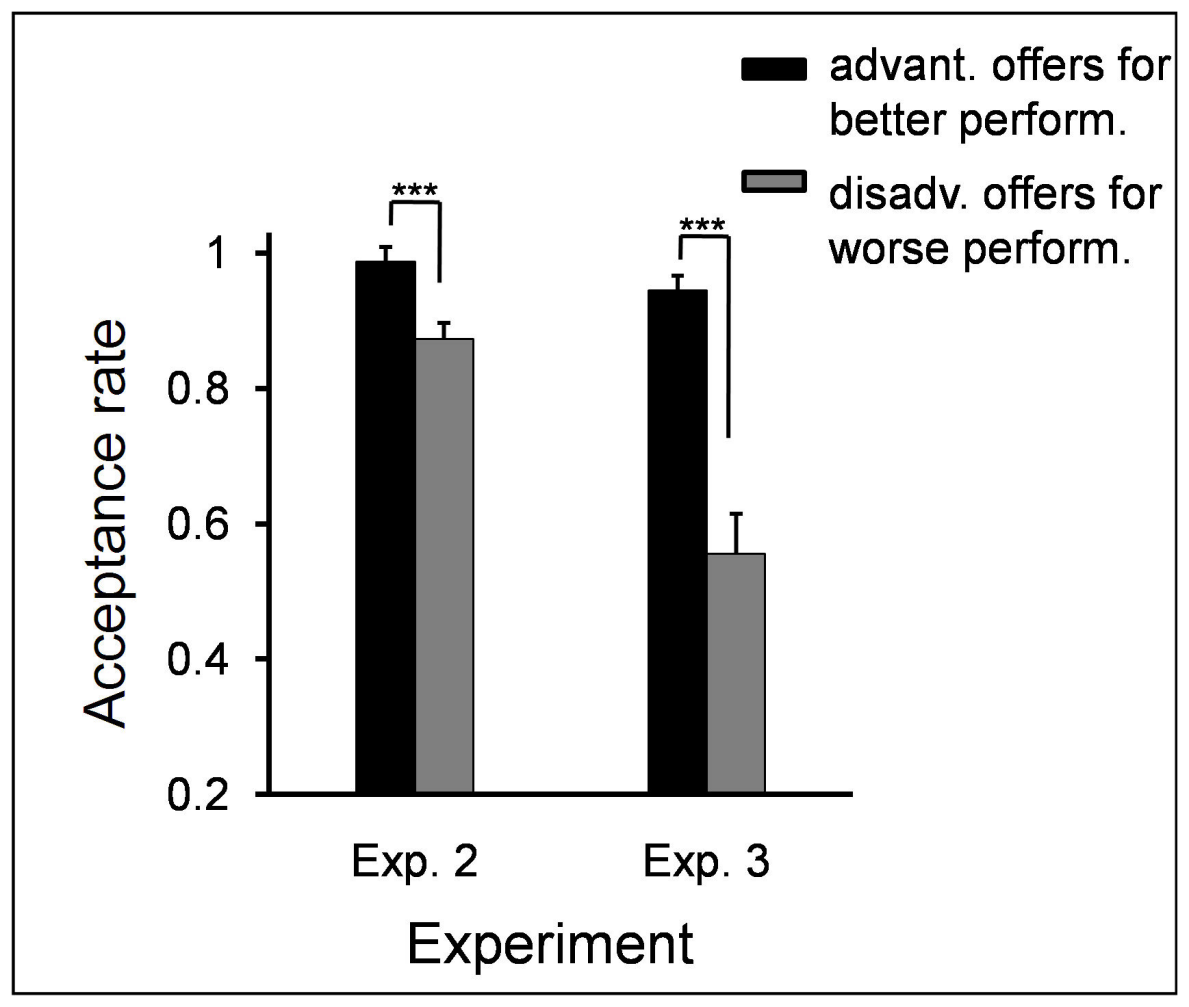
The acceptance rate of advantageous offers for better performance and disadvantageous offers for worse performance. Participants were more prone to accept advantageous offers for better performance, relative to disadvantageous offers for worse performance. Error bars indicate 1 SE. Advant. offers in better perform: advantageous offers for better performance; disadv. offers in worse perform: disadvantageous offers for worse performance.

In addition, a paired-samples t test was performed between acceptance rate of advantageous offers in the better-performance condition and that of disadvantageous offers in the worse-performance condition. This comparison was aimed to reveal whether participants strictly followed the same justice principle for both advantageous and disadvantageous offers [55]. The results revealed that the acceptance rate of advantageous offers (acceptance rate in 90:10 and 70:30 were collapsed) in the better-performance condition was higher than that of disadvantageous offers (acceptance rate in 10:90 and 30:70 were collapsed) in the worse-performance condition (*t*(69) = 4.76, *p* < .0005; see Figure 6).

#### Fairness Ratings

Two-way repeated measures ANOVA of Performance (better *vs.* even *vs.* worse) by Offer (90:10 *vs.* 70:30 *vs.* 50:50 *vs.* 30:70 *vs.* 10:90) yielded significant main effects of Performance (*F*(2,138) = 4.18, *p* < .05) and Offer (*F*(4,276) = 39.86, *p* < .0005) and a significant interaction between Performance and Offer (*F*(8,552) = 94.87, *p* < .0005). In general, the fairness rating was higher in the worse-performance condition compared with better-performance condition (*p* < .05) and was highest when the offer was 50:50 (*p* < .05).

**Figure 7:**
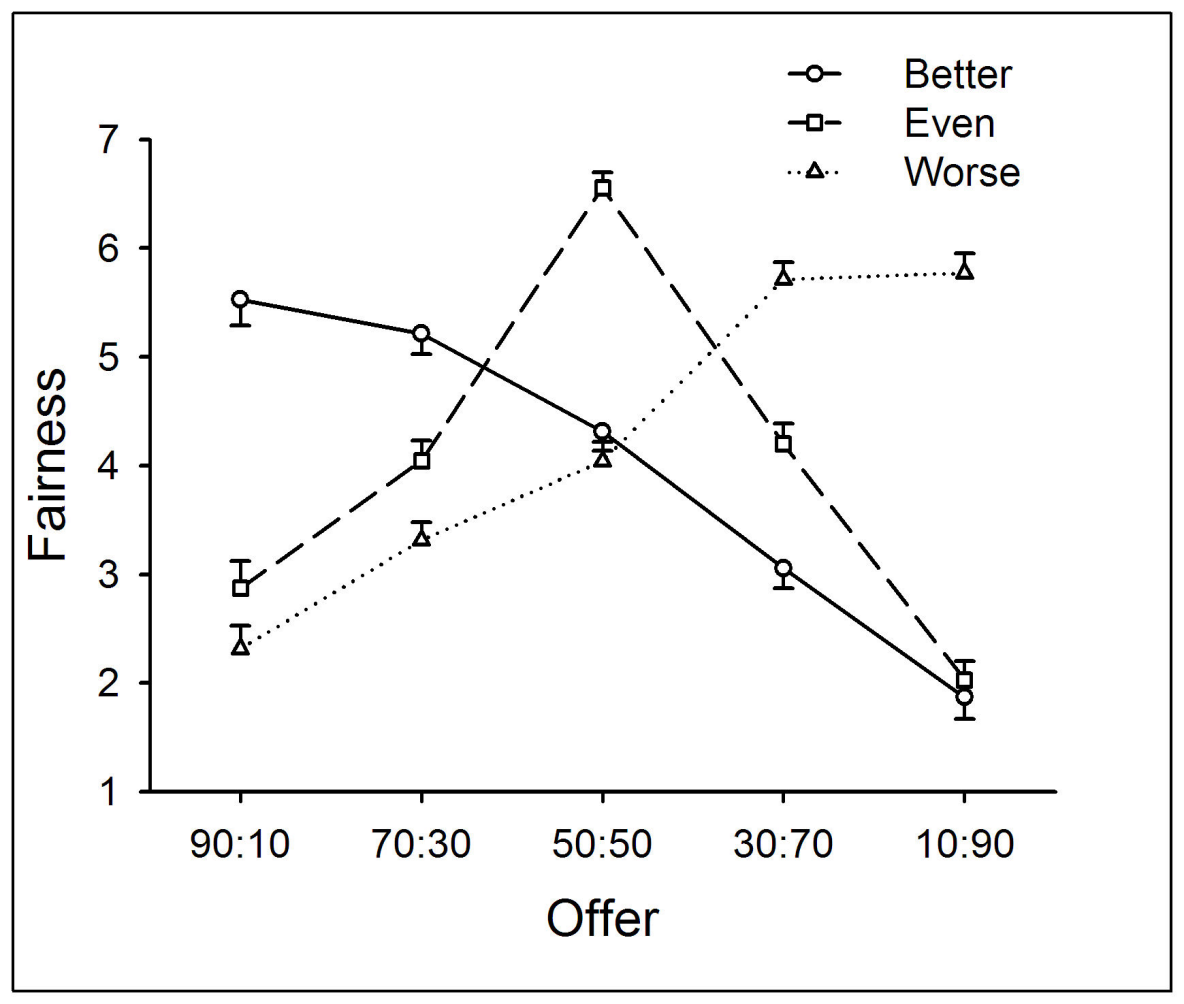
The fairness ratings in response to different kinds of offers within each performance condition in Experiment 2. The fairness rating was highest in the better-performance condition in response to advantageous offers; was highest in the even-performance condition in response to equal offers; and was highest in the worse-performance in response to disadvantageous offers. Error bars indicate 1 SE.

 Simple-effect analysis on the interaction revealed that, regarding the factor of Performance, for advantageous offers, the fairness rating was highest in the better-performance condition (*p* < .05); for equal offers, the rating was highest in the even-performance condition (*p* < .05); for disadvantageous offers, the rating was highest in the worse-performance condition (*p* < .05). Regarding the factor of Offer, in the better-performance condition, the fairness rating was highest for advantageous offers and lowest for disadvantageous offers (*p* < .05), whereas the reverse was true in the worse-performance condition (*p* < .05); in the even-performance condition, the rating reached its peak for equal offers (*p* < .05) (see Figure 7). 

### Discussion: Experiment 2

Experiment 2 replicated the major findings of Experiment 1. That is, participants’ allocation to their partners, as well as their acceptance rate, decreased as levels of performance increased. In addition, participants were more prone to reject advantageous unequal offers (i.e., 90:10) in the worse-performance condition than better-performance condition, indicating that such offers might be perceived “not so fair” when participants’ performance was worse than their partners. This finding suggests that earned entitlement also modulates individuals’ responses to unequal offers even when these offers are advantageous to them. However, responders’ acceptance rate of unequal offers was not completely motivated by justice principles; rather, they were motivated by self-interest. Namely, the acceptance rate of advantageous unequal offers in the better-performance condition was higher than that of disadvantageous unequal offers in the worse-performance condition.

Moreover, the fairness rating clearly showed the influence of performance on feelings of fairness. When participants performed better than their presumed partners, they rated advantageous unequal offers as the fairest offers; when their performance was even, equal split was judged as the fairest offers; finally, when participants’ performance was worse than their partners’, the fairest offers were disadvantageous unequal offers. These results suggest that the subjective ratings of fairness are determined by people’s sense of earned entitlement to the resources. Provided that the entitlement is biased between two players, fairness entails more than equality.

In Experiment 2, the fairness ratings and the behavioral responses to advantageous unequal offers showed distinct patterns. Specifically, although the subjective fairness rating to advantageous unequal offers was sensitive to levels of performance, participants accepted most of such offers in all performance conditions (see Figure 5). In our opinion, the repeated task design in Experiment 2 might be associated with this discrepancy. When participants played the role of UG-responders in multi-rounds, they might have considered not only the fairness level of the proposal in the current trial, but also the treatments they received in previous trials [26]. This view is in line with recent findings that people are less likely to conform to social norms when they see others violate a social norm, even if it is an unrelated social norm [56,57]. Alternatively, the advantageous offers in the worse-performance condition might lead participants to assume that offers were randomly generated rather than proposed by the opponent. This view was supported by the results that acceptance rates were high for both advantageous and disadvantageous unequal offers, indicating that people were prone to accept unequal offers due perhaps to the assumption that the opponent was not responsible for the offers (see also [37]). These confounding factors may have generated ceiling effects in acceptance rate (see Figure 5) and thereby attenuated the observed effects of entitlement on behavioral results. In order to eliminate these potential confounding factors, we employed one-shot version of the UG task in Experiment 3 to confirm the results of Experiments 1 and 2.

## Experiment 3

### Methods: Experiment 3

#### Ethics Statement

This study was completely approved by the Institutional Review Board (IRB) at Beijing Normal University. Written informed consents were collected for all participants.

#### Participants

Ninety college students aged 18–25 years (52 females; mean age 21.98 ± 1.93 years) participated in the experiment as paid volunteers. None of these students participated in Experiment 1 or 2.

#### Task Procedure

Participants were asked to complete the task with another anonymous person sitting in a different room with the door closed. In fact, no other people were playing with the participant. Similar with above two experiments, Experiment 3 contained a resource earning stage (the NET) and a following resource distribution stage (the UG and DG games).

In the first stage (see Text S2 for more information), participants completed 100 rounds of the NET on a personal computer. Each participant was told that he/she and his/her partner would together get a reward of 100 points if they give the correct answer in more than 100 rounds (in total). At the end of the NET, the computer “calculated” each person’s performance according to their accuracy rates and reaction times, which was then presented on the screen. In reality, the computer randomly assigned the participants to three groups regardless of their task performance. Accordingly, the sample was divided into the better-performance (16 females; mean age 22.00 ± 1.93 years), the even-performance (18 females; mean age 21.80 ± 1.90 years), and the worse-performance (18 females; mean age 22.13 ± 2.01 years) groups, each of which consisted of 30 participants. Independent-samples t tests revealed that these groups did not differ significantly in age (*p* > .05). For the better-performance group, the feedback presentation indicated that the participants’ contribution on the resource earning ranged between 75% and 85%; for the even-performance group, the contribution ranged between 45% and 55%; for the worse-performance group, the contribution ranged between 15% and 25%.

In the second stage, participants played one-shot UG (as proposers and responders) and DG (as proposers) by finishing three answer sheets (see supplemental materials). Nine kinds of potential offers were provided on the answer sheets, such that participants’ potential share ranged from 10 points (“you-10, other-90”) to 90 points (“you-90, other-10”). When playing as (UG and DG) proposers, participants made a proposal to their partner by selecting one offer from the options; when playing as UG responders, participants answered whether or not they would like to accept each kind of potential offer. This methodology, which is called the “strategy method,” has been widely used in lab studies for its advantage of increasing statistical power [58].

Following the formal tasks, participants were asked to rate the perceived levels of fairness (7-point scale) of each kind of offer. At the end of the experiment, all participants were completely debriefed about the deception and the object of the experiment.

#### Statistics

Chi-square tests were conducted to analyze participants’ acceptance rate in the UG responder session [58,59]. The factor of Offer was reconstructed as a three-level factor, such that the offers 90:10, 80:20, and 70:30 were labeled as “advantageous unequal offers;” the offers 60:40, 50:50, and 40:60 were labeled as “equal offers;” finally, the offers 30:70, 20:80, and 10:90 were labeled as “disadvantageous unequal offers.”

### Results: Experiment 3

#### Resources Allocation: Proposals

**Figure 8:**
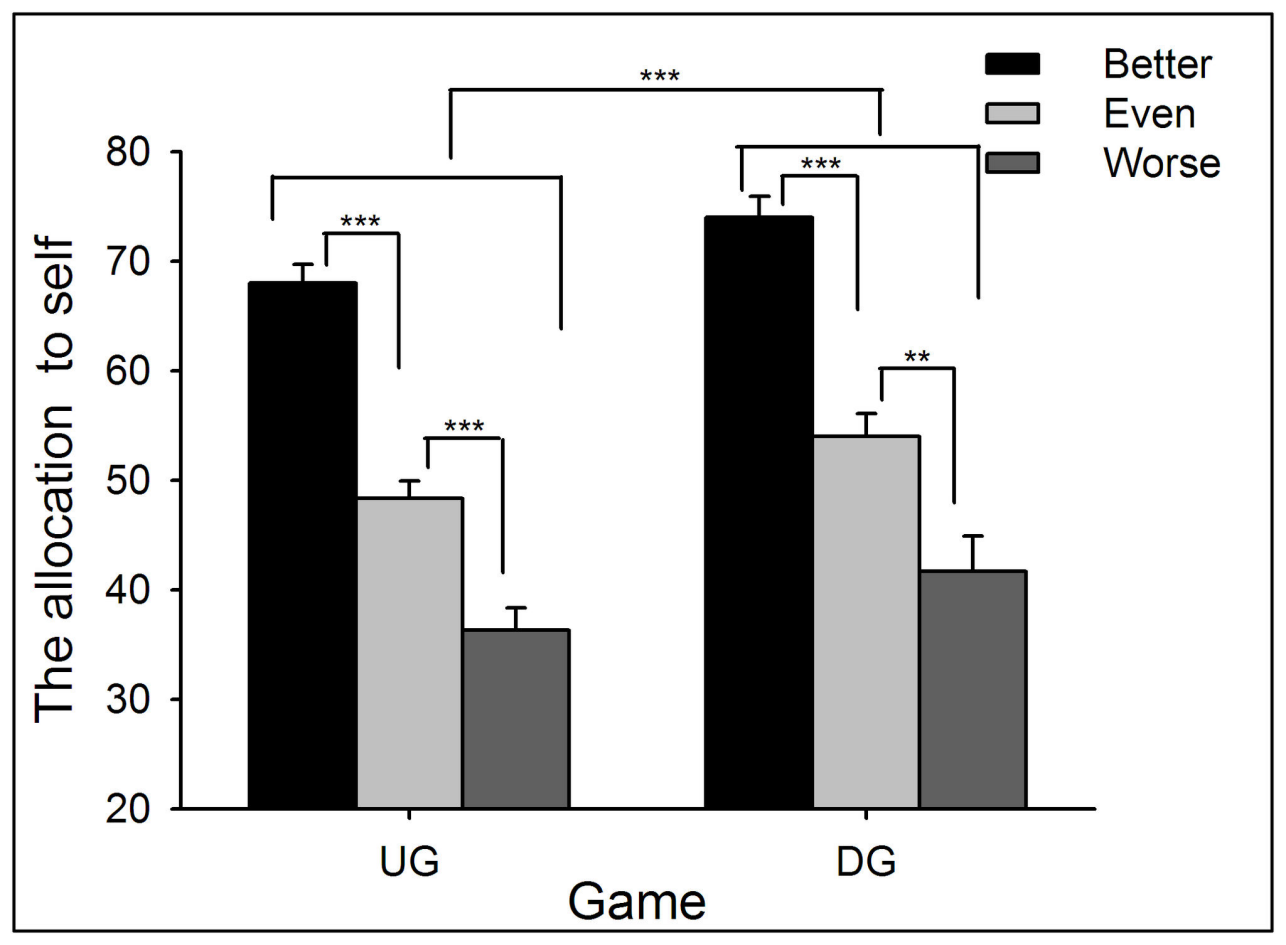
The average points participants allocated to themselves in the UG and DG in Experiment 3. Participants’ allocations to themselves were highest when their performance was better than partners and lowest in the worse-performance condition. In addition, participants allocated more to themselves in the DG than in the UG. Error bars indicate 1 SE. UG: ultimate game; DG: dictator game.

Two-way ANOVA of Performance (better *vs.* even *vs.* worse) by Game (UG *vs.* DG) yielded significant main effects of Performance (*F*(2,87) = 71.12, *p* < .0005) and Game (*F*(1,87) = 25.65, *p* < .0005). The patterns of these main effects were the same as Experiments 1 and 2 (see Figure 8 and Table S5). In addition, as a DG proposer, participants’ allocation to themselves in the better-performance condition was larger than that to the partner in the worse-performance condition (*t*(58) = 4.20, *p* < .0005).

#### Responses to Offers: Acceptance Rate

**Figure 9:**
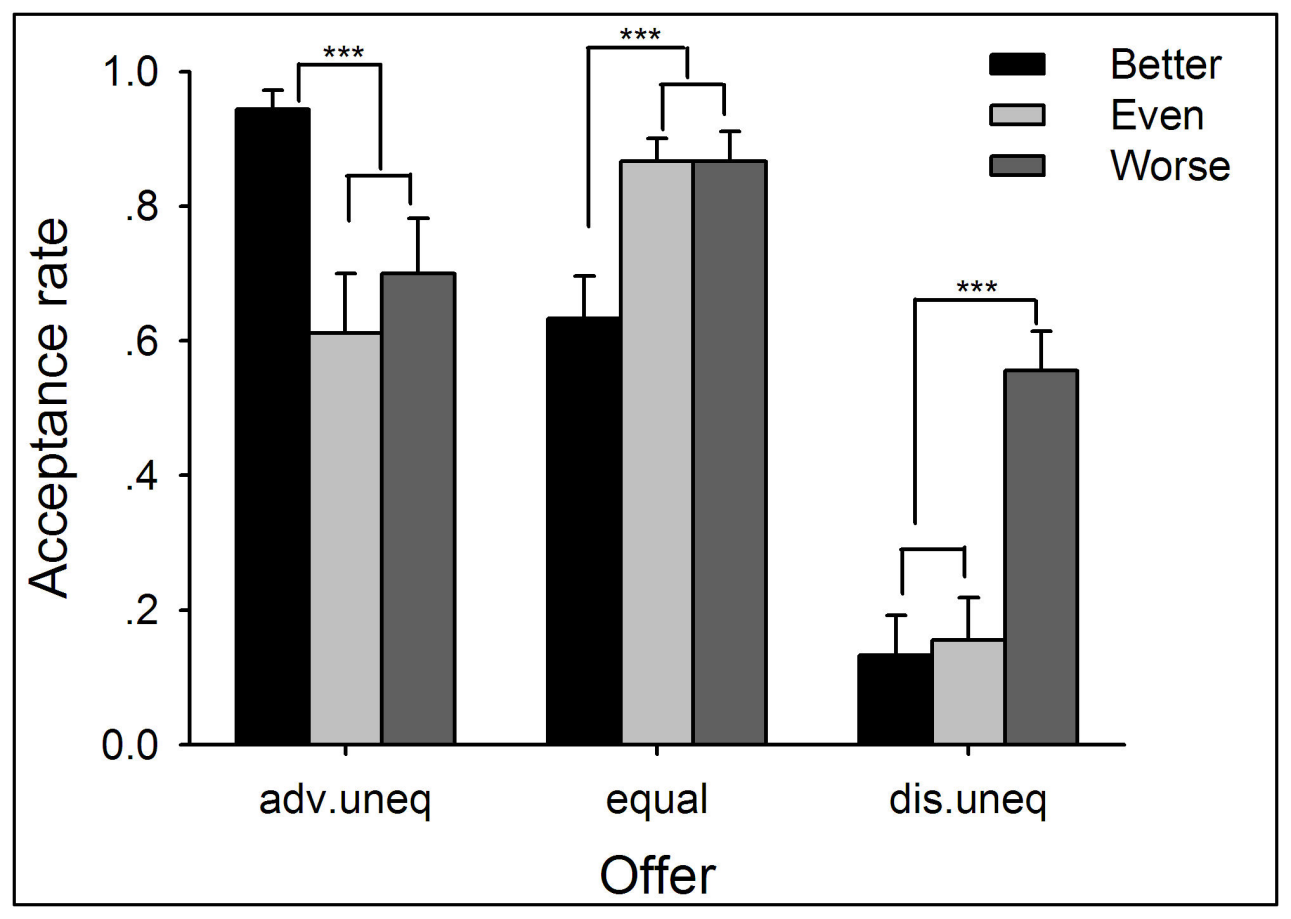
The average acceptance rates to different kinds of offers within each performance condition in Experiment 3. Participants were more likely to accept advantageous unequal offers when they performed better than their partner, and were more likely to accept disadvantageous unequal offers in the worse-performance condition. Error bars indicate 1 SE. Adv. uneq: advantageous unequal offers; dis.uneq: disadvantageous unequal offers.

The acceptance rate for advantageous unequal offers (90:10, 80:20, and 70:30) was higher in the better-performance condition (94.44%) than either the even- (61.11%; χ^2^(1) = 28.93, *p* < .0005) or the worse-performance condition (70%; χ^2^(1) = 18.40, *p* < .0005). Meanwhile, the acceptance rate for disadvantageous unequal offers (30:70, 20:80, and 10:90) was higher in the worse-performance condition (55.56%) than either the better- (13.33%; χ^2^(1) = 35.53, *p* < .0005) or the even-performance condition (15.56%; χ^2^(1) = 31.42, *p* < .0005). In short, participants were more likely to accept advantageous unequal offers when they performed better than their partner, and were more likely to accept disadvantageous unequal offers when their performance was worse (see Figure 9 and Table S6). Furthermore, the acceptance rate for equal offers (50:50) was lower in the better-performance condition (63.33%) than either the even- (86.67%; χ^2^(1) = 18.40, *p* < .0005) or the worse-performance condition (86.67%; χ^2^(1) = 18.40, *p* < .0005) (see Text S3 for more information). Finally, the acceptance rate of advantageous offers in the better-performance (94.44%) was higher than that of disadvantageous offers in the worse-performance (55.56%; χ^2^(1) = 36.30, *p* < .0005). These results were in accordance with the findings in Experiment 2 and implicated self-interest in the decision-making process.

#### Fairness Ratings

Two-way repeated measures ANOVA of Performance (better *vs.* even *vs.* worse) by Offer (advantageous *vs.* equal *vs.* disadvantageous) yielded significant main effects of Offer (*F*(8,696) = 52.95, *p* < .0005) and the interaction between Performance and Offer (*F*(16,696) = 22.26, *p* < .0005). The fairness rating was highest for equal offers (*p* < .05).

**Figure 10:**
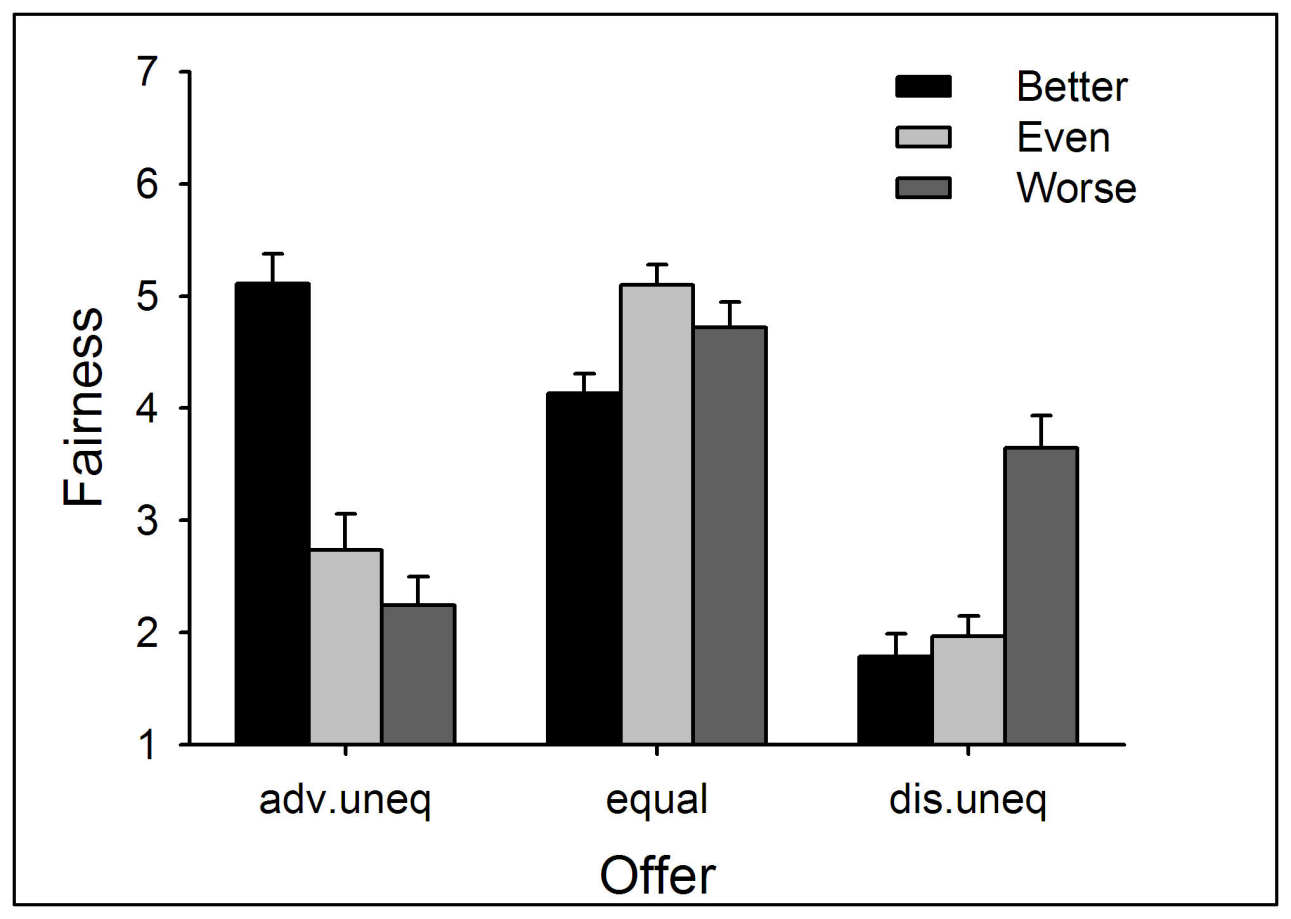
The fairness ratings in response to different kinds of offers within each performance condition in Experiment 3. The fairness rating was highest in the better-performance condition in response to advantageous offers; was highest in the even-performance condition in response to equal offers; and was highest in the worse-performance in response to disadvantageous offers. Error bars indicate 1 SE. Adv.uneq: advantageous unequal offers; dis.uneq: disadvantageous unequal offers.

 Simple-effect analysis on the interaction revealed that, regarding the factor of Performance, for advantageous offers, the fairness rating was highest in the better-performance condition; for equal offers, the rating was higher in the even- than the better-performance condition (*p* < .05); for disadvantageous offers, the rating was highest in the worse-performance condition (*p* < .05). Regarding the factor of Offer, in the better-performance condition, the fairness rating was highest for advantageous offers and lowest for disadvantageous offers (*p* < .05); in the even-performance condition, the rating was highest for equal offers (*p* < .05); in the worse-performance condition, the rating was highest for equal offers and lowest for advantageous offers (*p* < .05) (see Figure 10).

### Discussion: Experiment 3

Consistent with Experiments 1 and 2, the results of Experiment 3 suggested that participants’ allocation decisions, their responses to unequal offers, and their subjective ratings of offers were significantly influenced by performance. Most importantly, levels of performance modulated not only the responses to disadvantageous unequal offers, but also the responses to advantageous unequal offers, such that participants were less likely to accept advantageous offers when their performance was not better than their partners’. In addition, they rated such offers as fairer than equal-shares in the better-performance condition. It is therefore evidenced that fairness entails more than equality when levels of entitlement are biased between two parties.

## General Discussion

By providing a resource earning stage before resource allocation, we aimed to reveal distinct motivations that modulate individuals’ allocation as UG/DG proposers and their response to unequal offers as UG responders. First of all, the current study observed that earned entitlement significantly influenced both proposers’ and responders’ decisions. Proposers allocated more MUs to themselves and accepted more advantageous unequal offers in the better-performance condition than in the worse-performance condition. In accordance with participants’ behavioral decisions, it is clear that earned entitlement also influenced subjective feelings of fairness. In the better-performance condition, advantageous offers were considered as fairer proposals (see Figures 7 and 10), while in the worse-performance condition, the disadvantageous offers received higher fairness rating (see Figures 3, 7, and 10). These results indicate that fairness perception is flexible across different social contexts (see also 21,60, and preference for equitable outcomes described fairness perceptions only in the special case where earned entitlement between each side was the same. Furthermore, participants’ decisions were motivated by self-interest: a) MUs allocated to self in the DG were higher than those in the UG, b) MUs allocated to self in the better-performance condition were more than those to the partner in the worse-performance condition, and c) the acceptance rate of advantageous unequal offers for the better-performance condition was higher than that of disadvantageous unequal offers for the worse-performance condition. These results indicate that both preference for fairness and self-interest serve as important factors in the process of social decision-making. These distinct motivations are discussed in detail in the following sections.

### Preference for Fairness

Fairness is a crucial social norm that individuals obey during resource allocation. In the classical UG/DG task, “fairness” is modeled in its simplest form: equal shares. However, recent studies have indicated that the fairness judgment is sensitive to contextual cues such as earned entitlement [29]. In accord with recent evidence, the current study observed modulations of entitlement on both resource allocation and response to unequal offers.

Regarding participants’ decisions as UG/DG proposers, the MUs that participants allocated to themselves increased as a function of entitlement. These results are consistent with recent studies employing similar manipulation of entitlement. In these studies, it has been well demonstrated that proposers show very little preference for equality and keep most of resources when they have earned the entitlement [16,17,44,50,61]. More importantly, the current results reveal that proposers’ decisions are similar in the repeated and one-short versions of UG/DG tasks. Therefore, the current design can be directly combined with neuroimaging techniques (e.g., fMRI) to investigate the neural mechanisms underlying the effects of entitlement in resource allocation. Numerous neuroimaging studies have explored the neural correlates of resource allocation in the last decade [1], but the classical UG/DG tasks have been used in most such studies. Combining the current paradigm and advanced neuroimaging techniques will broaden the current literature on allocation behavior and related neural underpinnings.

Although a large body of literature regarding distributive justice has indicated the effects of entitlement in resources allocation, much less attention has been devoted to whether and how entitlement influences decisions in costly punishment. A prevalent view in the current literature is that costly punishment is driven by egalitarian motivation [2,62]. However, a robust finding observed in the current study was that participants showed enhanced tolerance to disadvantageous offers and were less likely to reject these offers (i.e., costly punishment) in the worse-performance condition. This result is consistent across repeated and one-shot games and suggests that costly punishments do not reflect a simple heuristics such as equal shares, but rather depend on considerations of earned entitlement.

Intriguingly, a similar pattern of the modulation of entitlement was also observed when participants responded to the advantageous offers: the acceptance rate of advantageous offers was higher in the better-performance condition than in the worse-performance condition. These findings provide support to the idea that individuals reject unequal but favorable offers [51–54] because people do not believe that they deserve such offers [15].

However, it should be noted that participants accepted most of the advantageous offers in the repeated UG, which was inconsistent with subjective ratings of fairness and decsions in the one-shot UG. These results may reflect that responders frequently observed others’ violations of fairness norms in the repeated UG (e.g., disadvantageous offers for better-performance). People are less likely to conform to fairness norms by rejecting favorable offers when they perceive that others violate social norms [56,57]. Alternatively, it is possible that the high acceptance rate of favorable offers may be due to the unrealistic settings in Experiment 2. For instance, the favorable unequal offers in the worse-performance condition might lead participants to assume that these offers were not proposed by the partner but were pre-programmed for participants and the partner. In this case, people are prone to accept unequal offers when they assume that the partner is not responsible for these offers [37,55]. This issue can be avoided by directly telling participants that offers are established by a random device rather than proposed by the partner. This procdeure may be a better choice when multiple rounds of interactions are needed (see also 55.

To sum up, the perception of fairness is sensitive to the sense of entitlement, an important contextual cue that constitutes distributive justice [29]. These results also fit with broader ideas of how social cues shape beliefs of fairness. Indeed, a large body of recent literature has revealed that the perception of fairness is subject to a variety of social cues, such as merit [63], expectation [20,64], intention [65], competition [66], and even implicit contextual manipulations [19]. These findings are in line with the current results and demonstrate that people apply flexible and adaptative justice principles to guide their decision-making in different social contexts. In other words, individuals integrate both pure preference for equality and social cues to form an adaptative fairness motivation.

### Self-interest

In addition to justice principles, self-interest also plays important roles in social decision-making and even can sometimes overcome fairness motivations. For instance, Civai et al. (2012) recently reported that acceptance rates showed no difference between advantageous and disadvantegous unequal offers when financial proposals were other-involved [55]. In contrast, the acceptance rate of advantegous unequal offers was much higher than that of disadvantegous unequal offers when fincianl proposals were self-involved, suggesting a self-serving bias.

Generally in accordance with previous studies regarding self-interest, two types of self-centered motivations were evidenced in the current study. The first was the stragetic motivation, such that participants allocated less MUs to themselves in the UG than those in the DG. These results reflect the fear of rejection and corresponding strategic motivations in the UG [33,40]. A recent developmental fMRI study revealed that the impulse control capacity is necessary to implement strategies in the UG task [40]. Another intriguing type of self-centered motivation is the self-serving bias established by both DG proposers and UG responders. Namely, the current findings indicate that people emphasize contextual cues (i.e., entitlement) to a larger extent in the better-performance condition relative to worse-performance condition. In other words, self-interest may serve as a crucial moderator to decide the extent to which contextual cues are biased when contextual aspects of fairness are integrated with equal-split aspect of fairness. People’s desire to appear fair to themselves and others may explain why people do not simply maximize their payoffs but rather apply such a subtle way [67,68]. This conjecture is in line with recent findings that people use a “incompletely-honest” strategy to balance payoffs and appearing honest [69,70].

In conclusion, the current results reveal that entitlement modulates behavioral decisions in resource allocation as well as subjective ratings of fairness. In this regard, fairness principles employed in the resource allocation are beyond egalitarian motivation. Similarly, earned entitlement also modulates individuals’ responses to disadvantageous unequal offers by enhancing tolerance to such offers when people perform worse than their partners. These results indicate that costly punishments are engaged to enforce the fairness principles that integrate both entitlement and preference for equality. Furthermore, the effects of entitlement also appears when participants respond to advantageous unequal offers, such that the acceptance rate of these offers is lower in the worse-performance condition than in the better-performance condition. These results suggest that advantageous offers are rejected because people feel that they do not deserve such offers. Finally, self-interest also manifests, such that earned entitlement is applied asymmetrically in a self-serving way to increase payoffs. That is to say, people take advantage of entitlement to act selfishly, although justice principles are implemented to some extent.

## Supporting Information

Figure S1 **An exemplar of stimuli presentation in the number estimation task.** One hundred red dots were presented on the screen, which was divided into equal left and right halves by a black line. In each trial, the number of red dots between the left and right sides always differed (i.e., varied between 40 and 60).
(TIF)

Figure S2 **Event sequence in an example trial when participants played the role of proposer in Experiments 1 and 2.** ITI: inter-trial interval.
(TIF)

Figure S3 **Event sequence in an example trial when participants played the role of responder in Experiments 1 and 2.** ITI: inter-trial interval.
(TIF)

Figure S4 **The proposal presentation in Experiment 1 when participants played the proposer role.** There were ten potential offers for participants to choose -90:10, 80:20, 70:30, 60:40, 50:50, 40:60, 30:70, 20:80, and 10:90. Participants were instructed to propose offers by pressing the corresponding button.
(TIF)

Figure S5 **The proposal used in Experiment 2 when participants played the proposer role.** There were five potential offers for participants to choose -90:10, 70:30, 50:50, 30:70, and 10:90.
(TIF)

Figure S6 **The average RT and ACC in the number estimation task in Experiment 1 when the participants played the role of proposer (error bars indicate 1 SE).**
(TIF)

Figure S7 **The average RT and ACC in the number estimation task in Experiment 1 when the participants played the role of responder (error bars indicate 1 SE).**
(TIF)

Figure S8 **The average RT and ACC in the number estimation task in Experiment 2 when the participants played the role of proposer (error bars indicate 1 SE).**
(TIF)

Figure S9 **The average RT and ACC in the number estimation task in Experiment 2 when the participants played the role of responder (error bars show 1 SE).**
(TIF)

Figure S10 **The RT and ACC in the number estimation task in Experiment 3 (error bars show 1 SE).**
(TIF)

Table S1 **The mean (with SD) MUs that participants allocated to themselves when playing the role of proposer.**
(DOC)

Table S2 **The mean (with SD) acceptance rate (%) in response to each kind of offer in different performance conditions.**
(DOC)

Table S3 **The mean (with SD) MUs that participants allocated to themselves when playing the role of proposer.**
(DOC)

Table S4 **The mean (with SD) acceptance rate (%) in response to each kind of offer in different performance conditions.**
(DOC)

Table S5 **The mean (with SD) MUs that participants allocated to themselves when playing the role of proposer.**
(DOC)

Table S6 **The mean (with SD) acceptance rate (%) in response to each kind of offer in different performance conditions.**
(DOC)

Text S1 **The instructions in Experiment 3.**
(DOC)

Text S2 **Participants’ performance in the number estimation task.**
(DOC)

Text S3 **Statistical analysis on accept rates of each offer in Experiment 3.**
(DOC)
