# Supplementary figures and images for: The Flexible Fairness: Equality, Earned Entitlement, and Self-Interest
Source: PLoS One. 2013 Sep 9;8(9):e73106. doi: 10.1371/journal.pone.0073106 (PMC3767679; doi:10.1371/journal.pone.0073106)

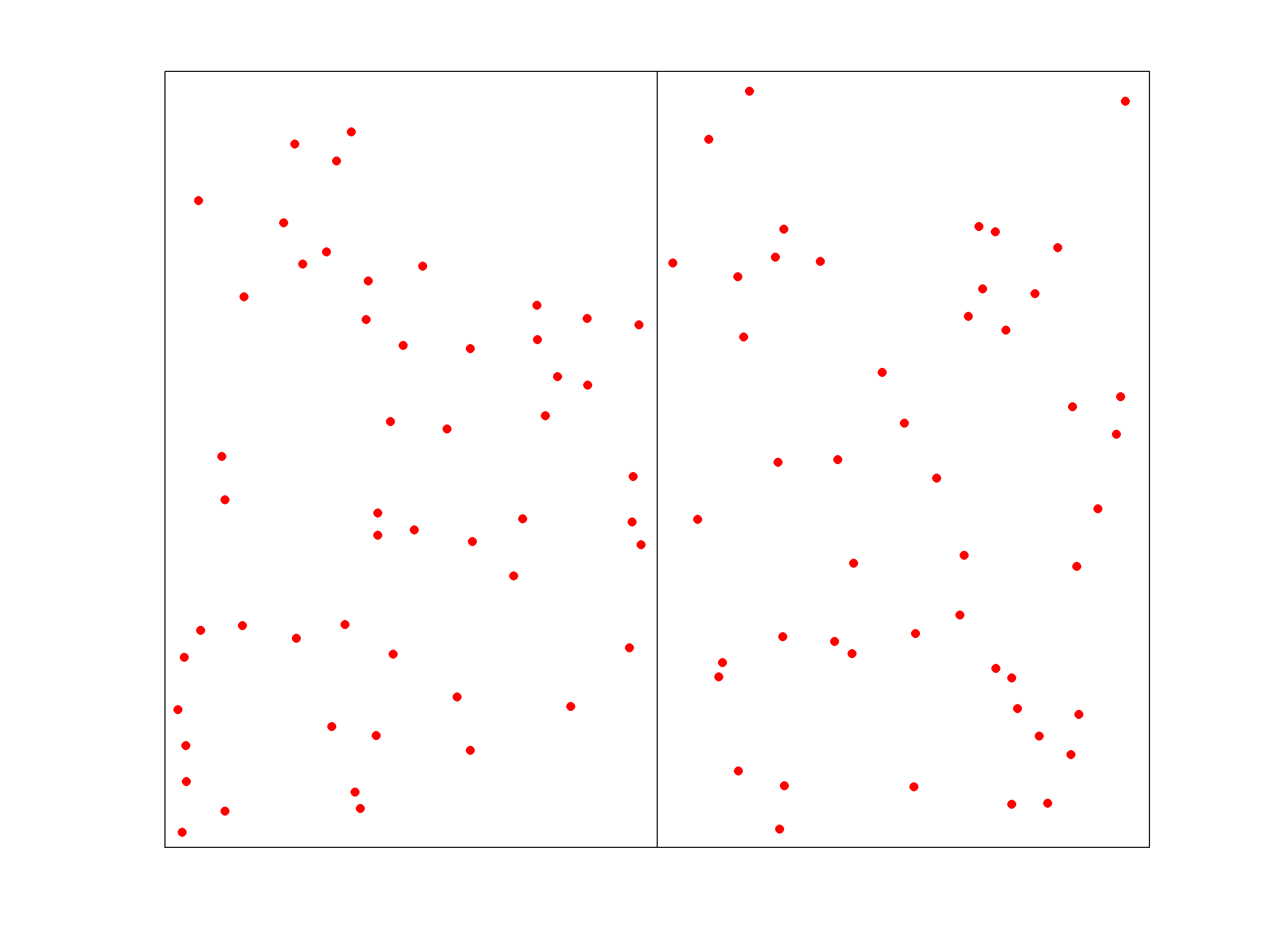

Supplement: Figure S1 — An exemplar of stimuli presentation in the number estimation task. One hundred red dots were presented on the screen, which was divided into equal left and right halves by a black line. In each trial, the number of red dots between the left and right sides always differed (i.e., varied between 40 and 60). (TIF) [file pone.0073106.s001.tif]

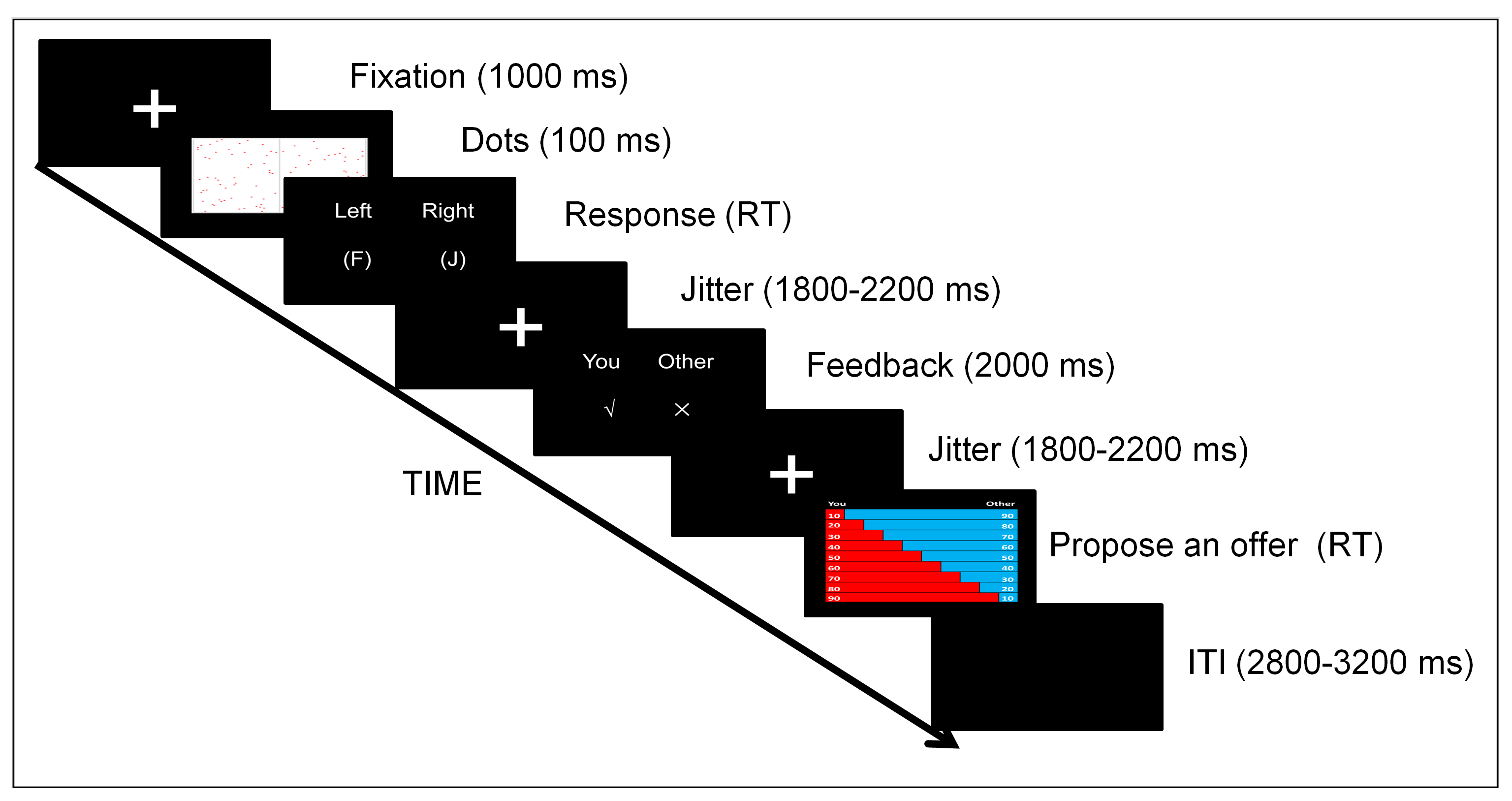

Supplement: Figure S2 — Event sequence in an example trial when participants played the role of proposer in Experiments 1 and 2. ITI: inter-trial interval. (TIF) [file pone.0073106.s002.tif]

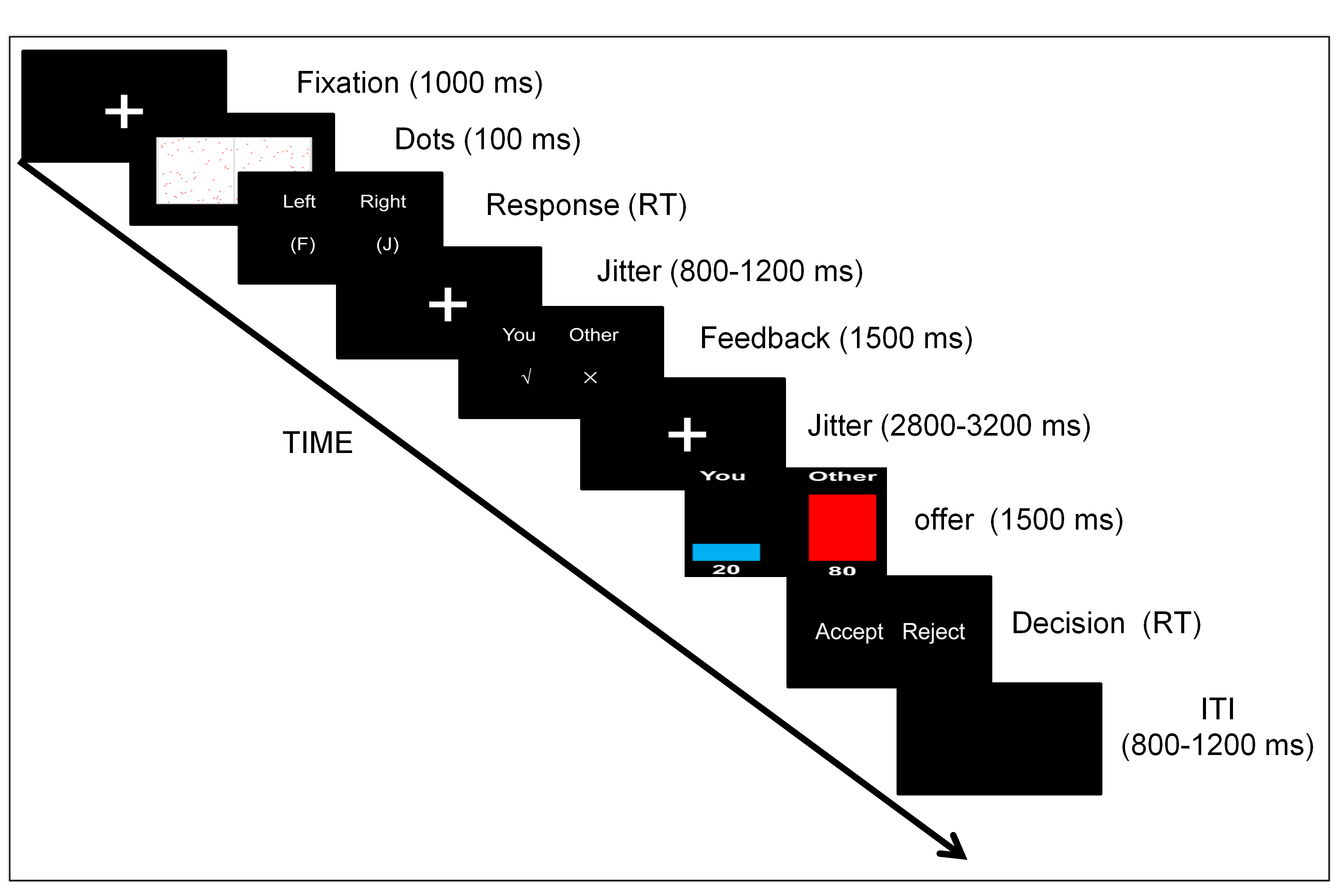

Supplement: Figure S3 — Event sequence in an example trial when participants played the role of responder in Experiments 1 and 2. ITI: inter-trial interval. (TIF) [file pone.0073106.s003.tif]

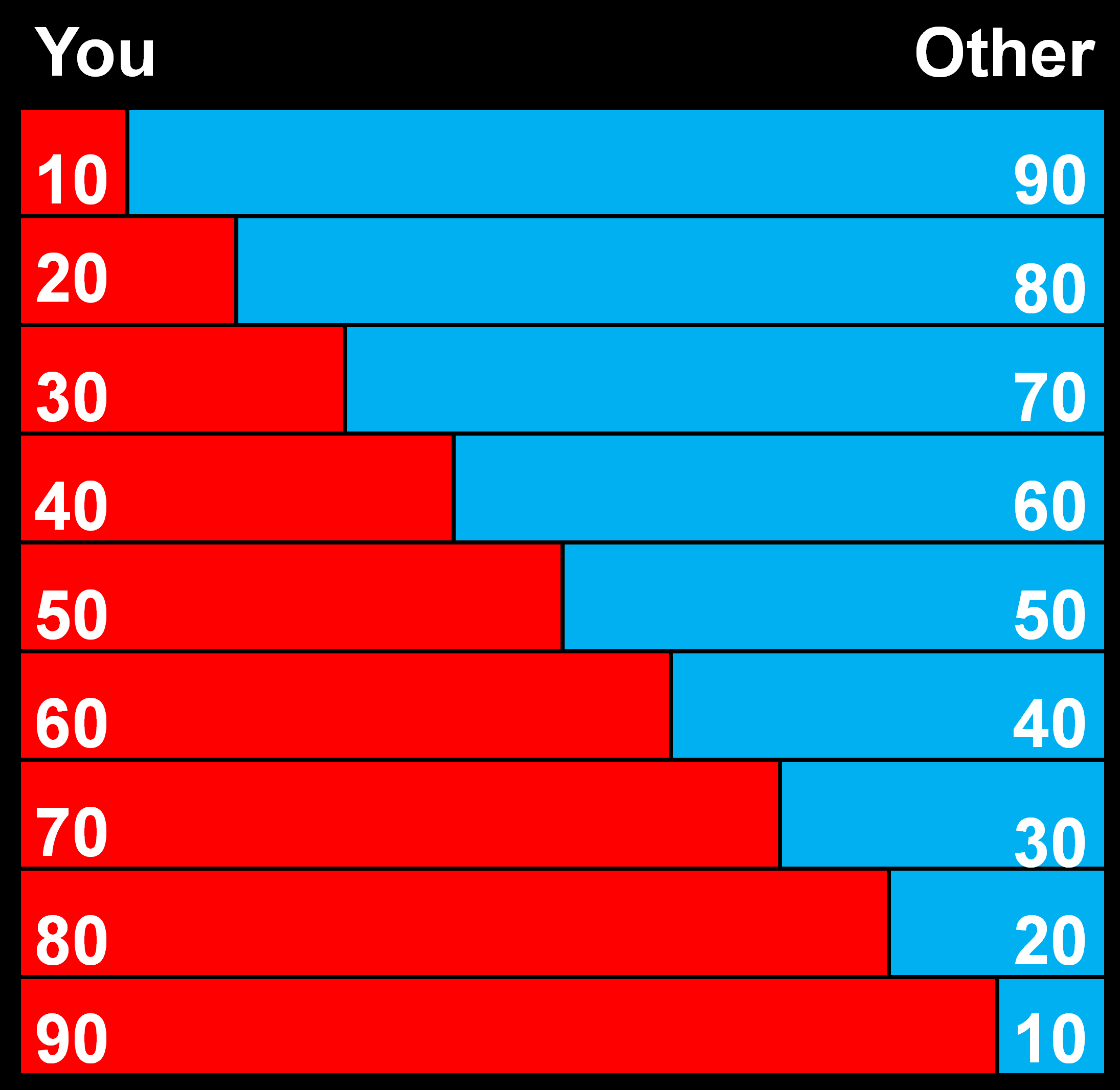

Supplement: Figure S4 — The proposal presentation in Experiment 1 when participants played the proposer role. There were ten potential offers for participants to choose -90:10, 80:20, 70:30, 60:40, 50:50, 40:60, 30:70, 20:80, and 10:90. Participants were instructed to propose offers by pressing the corresponding button. (TIF) [file pone.0073106.s004.tif]

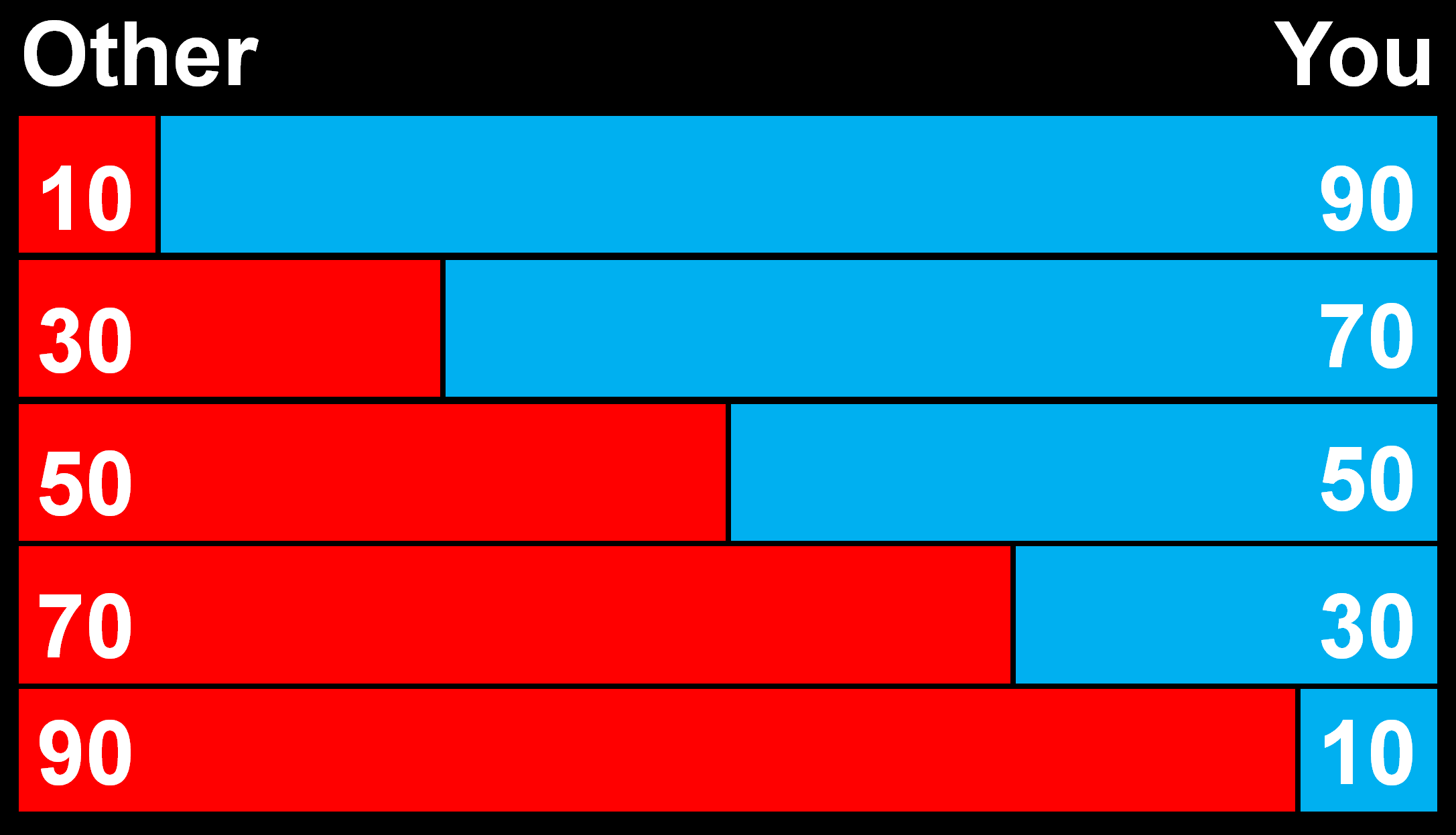

Supplement: Figure S5 — The proposal used in Experiment 2 when participants played the proposer role. There were five potential offers for participants to choose -90:10, 70:30, 50:50, 30:70, and 10:90. (TIF) [file pone.0073106.s005.tif]

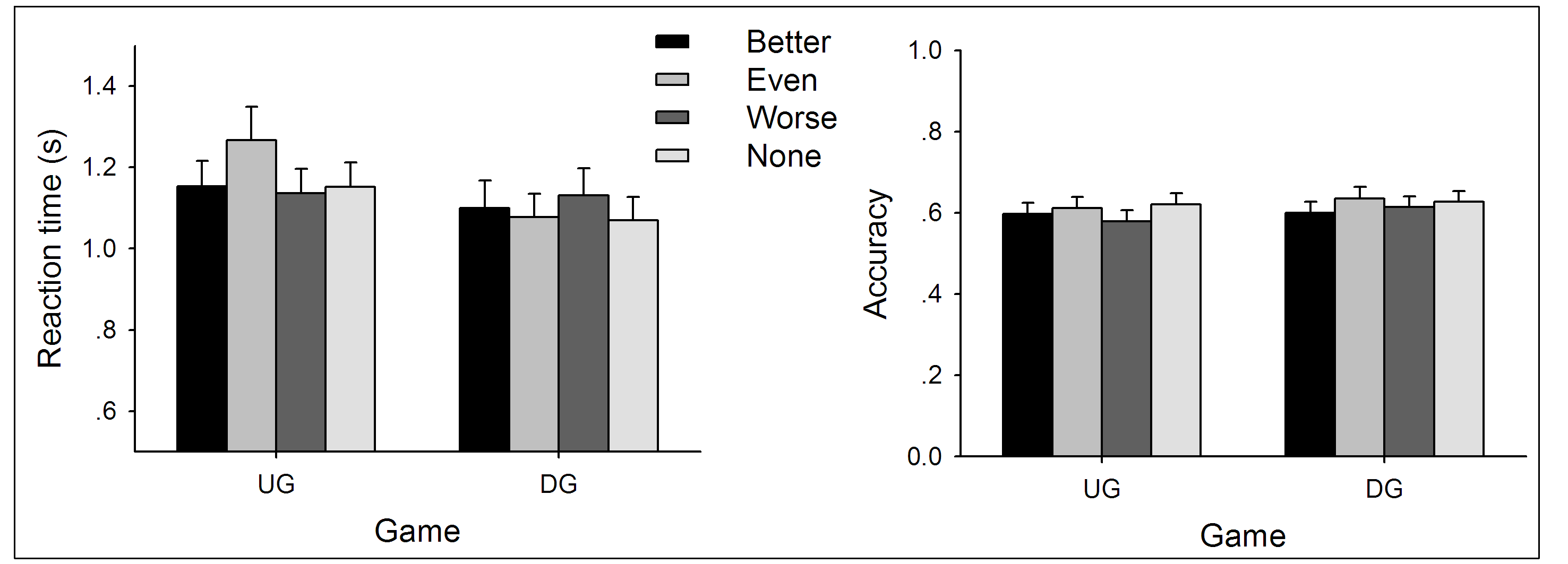

Supplement: Figure S6 — The average RT and ACC in the number estimation task in Experiment 1 when the participants played the role of proposer (error bars indicate 1 SE). (TIF) [file pone.0073106.s006.tif]

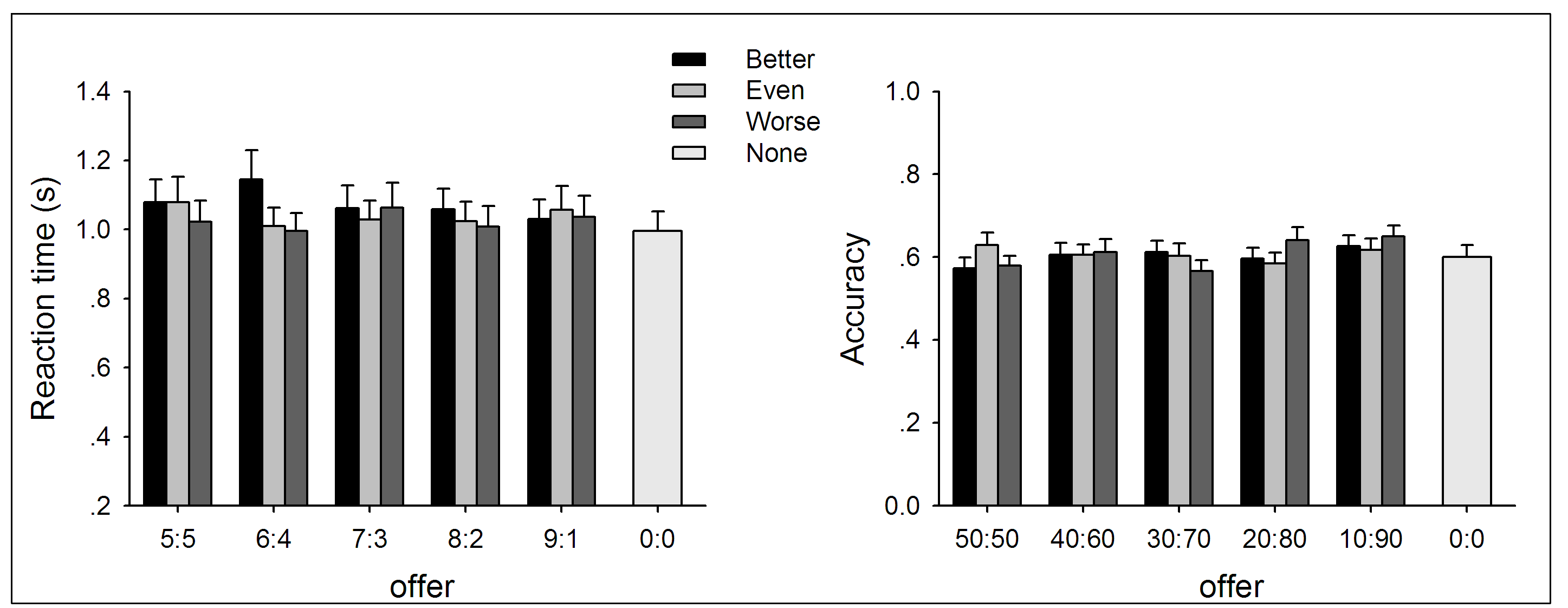

Supplement: Figure S7 — The average RT and ACC in the number estimation task in Experiment 1 when the participants played the role of responder (error bars indicate 1 SE). (TIF) [file pone.0073106.s007.tif]

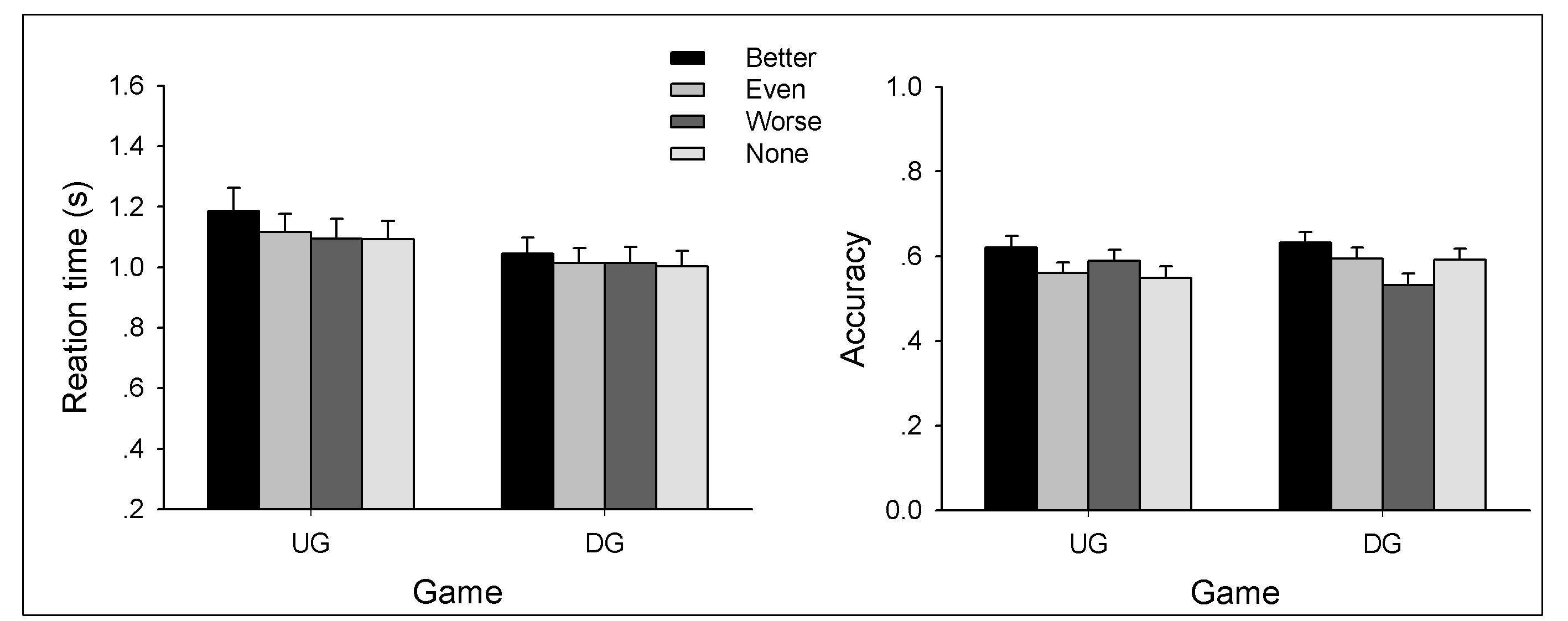

Supplement: Figure S8 — The average RT and ACC in the number estimation task in Experiment 2 when the participants played the role of proposer (error bars indicate 1 SE). (TIF) [file pone.0073106.s008.tif]

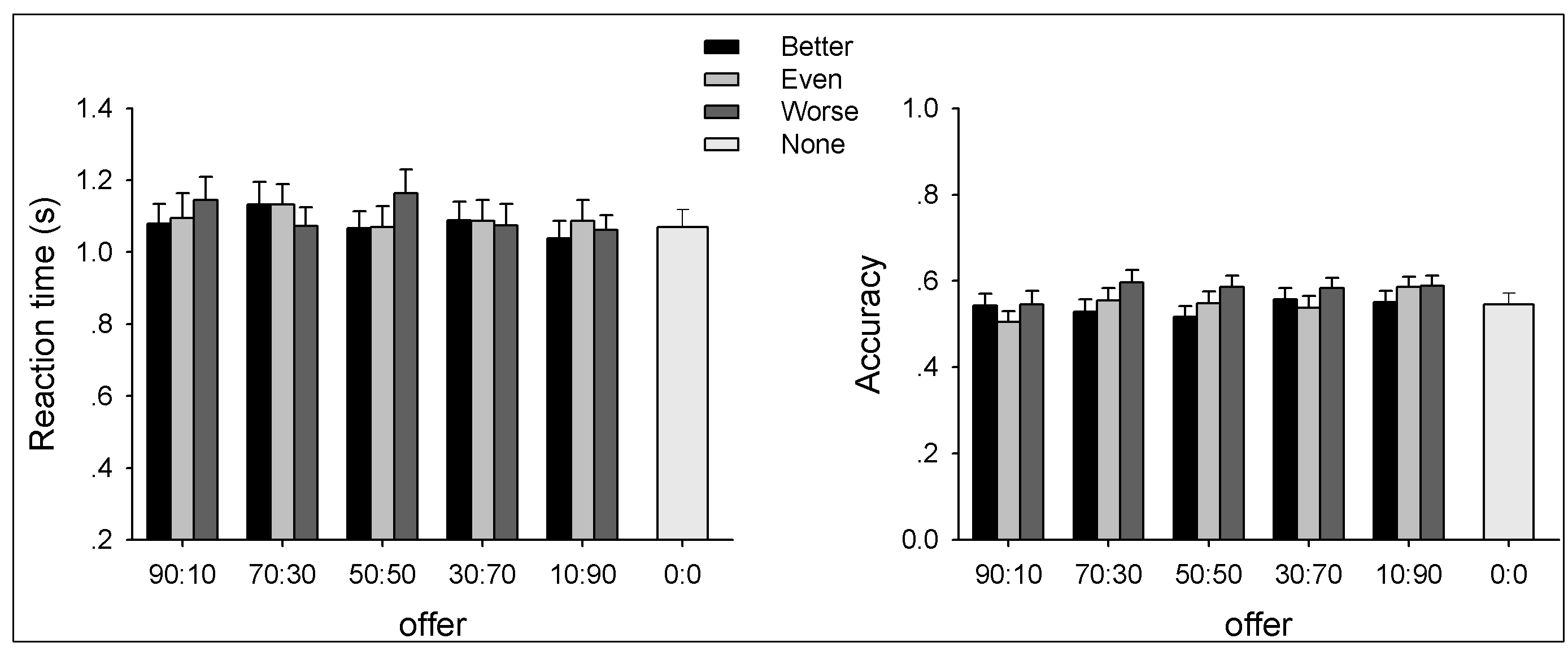

Supplement: Figure S9 — The average RT and ACC in the number estimation task in Experiment 2 when the participants played the role of responder (error bars show 1 SE). (TIF) [file pone.0073106.s009.tif]

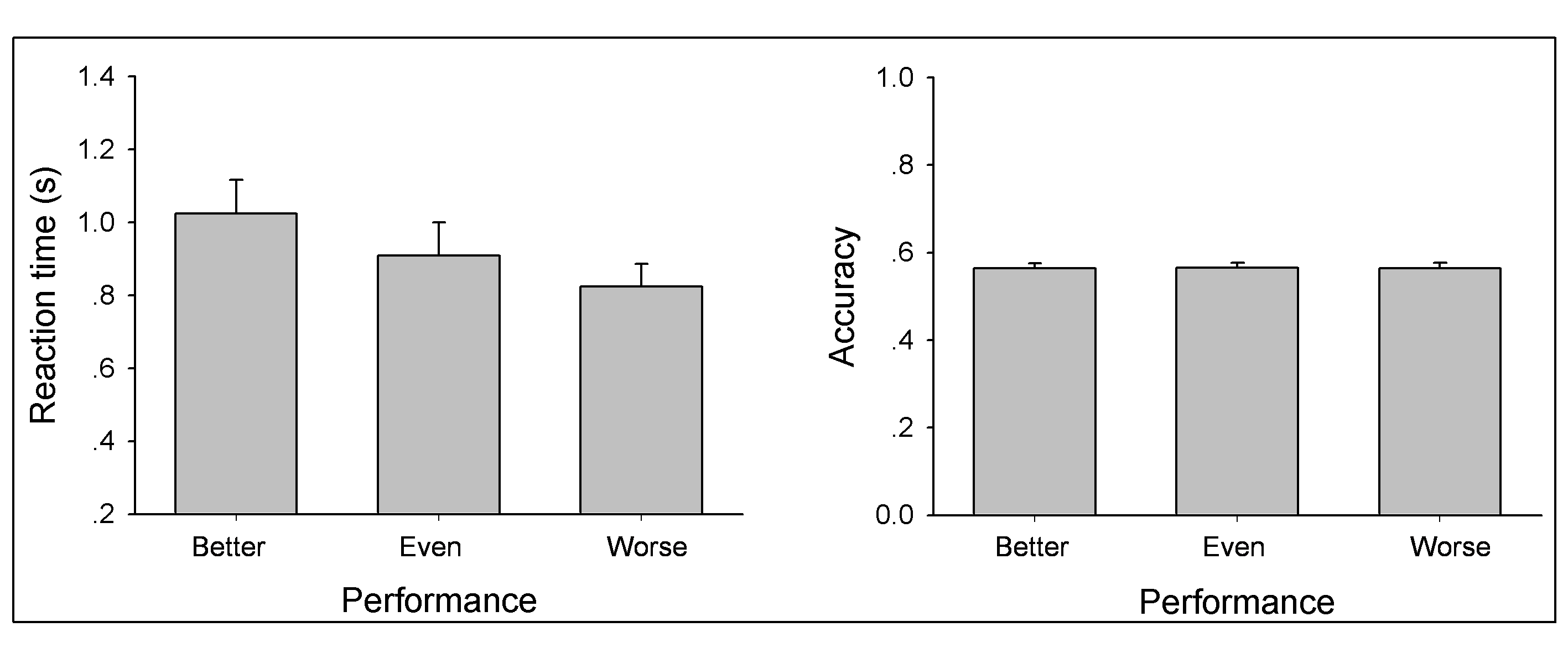

Supplement: Figure S10 — The RT and ACC in the number estimation task in Experiment 3 (error bars show 1 SE). (TIF) [file pone.0073106.s010.tif]
