# Supplementary material for: The Flexible Fairness: Equality, Earned Entitlement, and Self-Interest
Source: PLoS One. 2013 Sep 9;8(9):e73106. doi: 10.1371/journal.pone.0073106 (PMC3767679; doi:10.1371/journal.pone.0073106)
Supplement: Table S1 — The mean (with SD) MUs that participants allocated to themselves when playing the role of proposer. (DOC) [file pone.0073106.s011.doc]

| Participant’s allocation(MUs) | | | |
| --- | --- | --- | --- |
|  | Better | Even | Small |
| UG | 62.10 (10.98) | 51.88 (6.09) | 41.56 (12.90) |
| DG | 70.75 (15.37) | 57.31 (12.20) | 49.34 (16.98) |
