# Supplementary material for: The Flexible Fairness: Equality, Earned Entitlement, and Self-Interest
Source: PLoS One. 2013 Sep 9;8(9):e73106. doi: 10.1371/journal.pone.0073106 (PMC3767679; doi:10.1371/journal.pone.0073106)
Supplement: Table S2 — The mean (with SD) acceptance rate (%) in response to each kind of offer in different performance conditions. (DOC) [file pone.0073106.s012.doc]

| Participant’s accept rate | | | |
| --- | --- | --- | --- |
|  | Better | Even | Small |
| 50:50 | 97.31 (8.45) | 98.51 (6.34) | 99.70 (2.44) |
| 40:60 | 84.48 (31.11) | 89.25 (24.94) | 97.31 (12.98) |
| 30:70 | 66.27 (38.33) | 71.04 (35.89) | 94.03 (17.06) |
| 20:80 | 46.87 (42.00) | 49.25 (42.83) | 81.19 (31.89) |
| 10:90 | 30.75 (39.90) | 32.54 (40.88) | 61.19 (42.91) |
