# Supplementary material for: The Flexible Fairness: Equality, Earned Entitlement, and Self-Interest
Source: PLoS One. 2013 Sep 9;8(9):e73106. doi: 10.1371/journal.pone.0073106 (PMC3767679; doi:10.1371/journal.pone.0073106)
Supplement: Table S3 — The mean (with SD) MUs that participants allocated to themselves when playing the role of proposer. (DOC) [file pone.0073106.s013.doc]

| Participant’s allocation(MUs) | | | |
| --- | --- | --- | --- |
|  | Better | Even | Small |
| UG | 68.94 (14.88) | 53.23 (8.53) | 35.89 (15.76) |
| DG | 75.31 (14.76) | 60.40 (14.38) | 43.54 (19.15) |
