# Supplementary material for: The Flexible Fairness: Equality, Earned Entitlement, and Self-Interest
Source: PLoS One. 2013 Sep 9;8(9):e73106. doi: 10.1371/journal.pone.0073106 (PMC3767679; doi:10.1371/journal.pone.0073106)
Supplement: Table S4 — The mean (with SD) acceptance rate (%) in response to each kind of offer in different performance conditions. (DOC) [file pone.0073106.s014.doc]

| Participant’s accept rate | | | |
| --- | --- | --- | --- |
|  | Better | Even | Small |
| 90:10 | 98.29 (8.84) | 96.29 (9.20) | 90.29 (24.49) |
| 70:30 | 99.14 (5.31) | 98.86 (5.78) | 95.43 (13.26) |
| 50:50 | 95.43 (14.91) | 99.43 (4.78) | 98.00 (9.10) |
| 30:70 | 68.86 (35.33) | 81.71 (27.19) | 95.14 (16.48) |
| 10:90 | 42.86 (39.16) | 52.29 (39.90) | 79.43 (30.07) |
