# Supplementary material for: The Flexible Fairness: Equality, Earned Entitlement, and Self-Interest
Source: PLoS One. 2013 Sep 9;8(9):e73106. doi: 10.1371/journal.pone.0073106 (PMC3767679; doi:10.1371/journal.pone.0073106)
Supplement: Table S5 — The mean (with SD) MUs that participants allocated to themselves when playing the role of proposer. (DOC) [file pone.0073106.s015.doc]

| Participant’s allocation(MUs) | | | |
| --- | --- | --- | --- |
|  | Better | Even | Small |
| UG | 68.00 (9.25) | 48.33 (8.74) | 36.33 (10.98) |
| DG | 74.00 (10.37) | 54.00 (11.32) | 41.67 (17.63) |
