# Supplementary material for: The Flexible Fairness: Equality, Earned Entitlement, and Self-Interest
Source: PLoS One. 2013 Sep 9;8(9):e73106. doi: 10.1371/journal.pone.0073106 (PMC3767679; doi:10.1371/journal.pone.0073106)
Supplement: Table S6 — The mean (with SD) acceptance rate (%) in response to each kind of offer in different performance conditions. (DOC) [file pone.0073106.s016.doc]

| 70:30 | 100.00 (0.00) | 63.33 (49.01) | 73.33 (44.98) |
| --- | --- | --- | --- |
| 60:40 | 83.33 (37.90) | 93.33 (25.37) | 73.33 (44.98) |
| 50:50 | 76.67 (43.01) | 100.00 (0.00) | 93.33 (25.37) |
| 40:60 | 30.00 (46.61) | 66.67 (47.94) | 93.33 (25.37) |
| 30:70 | 16.67 (37.90) | 20.00 (40.68) | 83.33 (37.90) |
| 20:80 | 13.33 (34.57) | 13.33 (34.57) | 66.67 (47.95) |
| 10:90 | 10.00 (30.51) | 13.33 (34.57) | 16.67 (37.90) |
