# Supplementary material for: The Flexible Fairness: Equality, Earned Entitlement, and Self-Interest
Source: PLoS One. 2013 Sep 9;8(9):e73106. doi: 10.1371/journal.pone.0073106 (PMC3767679; doi:10.1371/journal.pone.0073106)
Supplement: Text S1 — The instructions in Experiment 3. (DOC) [file pone.0073106.s017.doc]

1. **Participants’ performance in the number estimation task.**
   1. Experiment 1.

*2.1.1. The UG/DG proposer phase*

Two-way ANOVA of performance (better *vs.* even *vs.* worse *vs*. none) by game (UG *vs.* DG) on reaction time (RT) yielded a significant effect of game (*F* (1, 66) = 4.57, *p* = .036), such that participants responded faster in the DG than UG. The main effect of performance (*F* (3, 198) = 1.39, *p* = .25) did not reach significance. The Performance × Game interaction was significant (*F* (3, 198) = 2.80, *p* = .049), such that participants responded faster in the DG than UG in the even-performance condition (*p* < .05).

Two-way ANOVA on accuracy (ACC) did not yield any significant effect: performance (*F* (3, 198) = .69, *p* = .56), game (*F* (1, 66) = 1.10, *p* = .30), or the interaction (*F* (3, 198) = .17, *p* = .92).

*2.1.2. The UG responder phase*

Two-way repeated measures ANOVA of performance (better *vs.* even *vs.* worse) by offer (10:90 *vs.* 20:80 *vs.* 30:70 *vs.* 40:60 *vs.* 50:50) on RT did not yield any significant effect: performance (*F* (2, 132) = 2.38, *p* = .10), offer (*F* (4, 264) = .30, *p* = .88), or the interaction (*F* (8, 528) = .94, *p* = .49).

Two-way ANOVA on ACC did not reveal any significant effect: performance (*F* (2, 132) = .11, *p* = .90), offer (*F* (4, 264) = 1.00, *p* = .41), or the interaction (*F* (8, 528) = .97, *p* = .46).

- 1. Experiment 2.

*2.2.1. The UG/DG proposer phase*

Two-way ANOVA of performance (better *vs.* even *vs.* worse *vs*. none) by game (UG *vs.* DG) on RT yielded a significant effect of game (*F*(1, 69) = 7.51, *p* < .01), such that participants responded faster in the DG than UG. Neither the main effect of performance (*F*(3, 207) = 2.56, *p* = .07) nor the interaction effect (*F*(3, 207) = .84, *p* = .47) reached significance.

Two-way ANOVA on ACC did not yield any significant effect: performance (*F* (3, 207) =2.32, *p* = .08), game (*F* (1, 69) =.24, *p* = .62), or the interaction (*F* (3, 207) = 1.55, *p* =.20).

*2.2.2. The UG responder phase*

Two-way repeated measures ANOVA of performance (better *vs.* even *vs.* worse) by offer (90:10 *vs.* 70:30 *vs.* 50:50 *vs.* 30:70 *vs.* 10:90) on RT did not yield any significant effects: performance (*F* (2, 138) = .87, *p* = .42), offer (*F* (4, 276) = 1.16, *p* = .33), or the interaction (*F* (8, 552) = 1.16, *p* = .32).

Two-way ANOVA on ACC did not reveal any significant effects: performance (*F* (2, 138) = 3.08, *p* = .051), offer (*F* (4, 276) = 1.13, *p* = .34), or the interaction (*F* (8, 552) = .52, *p* = .84).

- 1. Experiment 3.

One-way repeated measures ANOVA of performance (better *vs.* even *vs.* worse) on RT (*F* (2, 87) = .01, *p* = .99) and ACC (*F* (2, 87) = 1.49, *p* = .23) did not yield any significant effect.
