# Supplementary material for: The Flexible Fairness: Equality, Earned Entitlement, and Self-Interest
Source: PLoS One. 2013 Sep 9;8(9):e73106. doi: 10.1371/journal.pone.0073106 (PMC3767679; doi:10.1371/journal.pone.0073106)
Supplement: Text S2 — Participants’ performance in the number estimation task. (DOC) [file pone.0073106.s018.doc]

The instructions in Experiment 3 (see also Yamagishi et al., 2009)

*See below for the translated version of the instruction. The original text is written in Standard Chinese (Mandarin).*

*The first page of the instruction*

Thank you for participating in this experiment! This experiment is a part of our decision-making research. The data will not be used except for research purposes. Participants’ personal information will be omitted in final reports.

**Introduction of Experiment:** You and another student who will play as your “partner” will participate in this experiment anonymously. You do not have to directly communicate with your partner either during or after the experiment.

In this experiment, both you and your partner will finish a number estimation task individually. The task consists of 100 rounds. **At the end of the task, if the sum of you and your partner’s total correct responses is higher than 100, then both of you will receive a reward together.** In that case, the system will automatically compute the personal contribution of you and your partner in the task. **This computation is based on:（1）the number of correct responses, and（2）the response time (the faster, the better).** The results will then be presented on the screen.

The experimenter that you directly communicate with will have nothing to do with your decisions. If you have any concerns about the instruction, feel free to ask the experimenter. The number estimation task will begin after everyone is ready.

*The second page of the instruction*

**The number estimation task is over now. The allocation stage of the experiment will be next. Please read the following instruction carefully and make your decisions.**

You and your partner have won the reward. Please confirm that you know your contribution in the number estimation task. In the next stage, the reward will be distributed between you and your partner. **At the end of experiment, you will be paid according to the amount of money units you receive.**

*The instruction for UG proposers*

- **Please decide how to allocate the 100 money units.**
- Your partner will decide to accept or reject your offer.

| Allocation | | Your decision |
| --- | --- | --- |
| Keep for yourself | Leave to your partner |
| 90 MU | 10 MU |  |
| 80 MU | 20 MU |  |
| 70 MU | 30 MU |  |
| 60 MU | 40 MU |  |
| 50 MU | 50 MU |  |
| 40 MU | 60 MU |  |
| 30 MU | 70 MU |  |
| 20 MU | 80 MU |  |
| 10 MU | 90 MU |  |

- To make a proposal, please draw a mark in the blank space.
- Your partner will decide to accept or reject your offer.
- If your partner accepts your offer**, then the two of you will receive the reward accordingly.**
- If your partner rejects your offer, **neither of you will receive anything.**

*The instruction for DG proposers*

- **Please decide how to allocate the 100 money units.**
- Since your partner could not reject your offer, the reward will be divided according to your proposal.

| Allocation | | Your decision |
| --- | --- | --- |
| Keep for yourself | Leave to your partner |
| 90 MU | 10 MU |  |
| 80 MU | 20 MU |  |
| 70 MU | 30 MU |  |
| 60 MU | 40 MU |  |
| 50 MU | 50 MU |  |
| 40 MU | 60 MU |  |
| 30 MU | 70 MU |  |
| 20 MU | 80 MU |  |
| 10 MU | 90 MU |  |

- To make a proposal, please draw a mark in the blank space.
- Your partner could not reject your offer, and the reward will be divided according to your proposal.

*The instruction for UG responders*

| Allocation | | Your partner’s decision |
| --- | --- | --- |
| Keep for him/herself | Leave to you |
| 90 MU | 10 MU |  |
| 80 MU | 20 MU |  |
| 70 MU | 30 MU |  |
| 60 MU | 40 MU |  |
| 50 MU | 50 MU |  |
| 40 MU | 60 MU |  |
| 30 MU | 70 MU |  |
| 20 MU | 80 MU |  |
| 10 MU | 90 MU |  |

- Your partner will make a proposal by selecting one of ten potential offers from the table on the right side.
- Please indicate your response (accept/reject) to each kind of offers in the next table (see below).

**Your decision**

| Allocation | | Your decision |
| --- | --- | --- |
| Keep for him/herself | Leave to you |
| 90 MU | 10 MU |  |
| 80 MU | 20 MU |  |
| 70 MU | 30 MU |  |
| 60 MU | 40 MU |  |
| 50 MU | 50 MU |  |
| 40 MU | 60 MU |  |
| 30 MU | 70 MU |  |
| 20 MU | 80 MU |  |
| 10 MU | 90 MU |  |

- Please indicate your decision to **each kind of potential offer** by writing a “√” mark next to the offer (if you accept it) or writing a “×” mark (if you reject it).
- After your partner gives his/her proposal, the experimenter will get your decision from this table.
  - If you accept the offer, **then the two of you will receive the reward accordingly.**
  - If your reject the offer, **neither of you will receive anything.**

*The final page of the instruction*

The allocation stage is over now. Please put all the answer sheets into the envelope, and tell the experimenter that you have finished.

Another experimenter will then check the answer sheets from both of you. You will be paid according to you and your partner’s decisions.

**Reference**

Yamagishi, T., Horita, Y., Takagishi, H., Shinada, M., Tanida, S., & Cook, K. S. (2009). The private rejection of unfair offers and emotional commitment. *Proc Natl Acad Sci U S A, 106*(28), 11520-11523.
