# Supplementary material for: The Flexible Fairness: Equality, Earned Entitlement, and Self-Interest
Source: PLoS One. 2013 Sep 9;8(9):e73106. doi: 10.1371/journal.pone.0073106 (PMC3767679; doi:10.1371/journal.pone.0073106)
Supplement: Text S3 — Statistical analysis on accept rates of each offer in Experiment 3. (DOC) [file pone.0073106.s019.doc]

For the 90:10 offer, the accept rate was higher in the better-performance condition (86.67%) than the even- (60%; χ^2^ (1) = 5.46, *p* < .05) and worse-performance condition (66.67%; χ^2^ (1) = 3.35, *p* = .067).

For the 80:20 offer, the accept rate was higher in the better-performance condition (96.67%) than the same- (60%; χ^2^ (1) = 11.88, *p* < .005) and worse-performance condition (70%; χ^2^ (1) = 7.68, *p* < .01).

For the 70:30 offer, the accept rate was higher in the better-performance condition (100%) than the even- (63.33%; χ^2^ (1) = 13.67, *p* < .0005) and worse-contribution condition (73.33%; χ^2^ (1) = 9.23, *p* < .005).

For the 60:40 offer, the accept rate was higher in the even-performance condition (93.33%) than the worse-performance condition (73.33%; χ^2^ (1) = 4.32, *p* < .05).

For the 50:50 offer, the accept rate was lower in the better-performance condition (76.67%) than the even- (100%; χ^2^ (1) = 7.93, *p* < .01) and worse-performance condition (93.33%; χ^2^ (1) = 3.27, *p* = .07).

For the 40:60 offer, the accept rate was higher in the worse-performance (93.33%) condition than both the even- (66.67%; χ^2^ (1) = 6.67, *p* < .05) and better-performance condition (30%; χ^2^ (1) = 25.45, *p* < .0005), while the accept rate in the even-performance condition was higher than the better-performance condition (χ^2^ (1) = 8.08, *p* < .01).

For the 30:70 offer, the accept rate was higher in the worse-performance condition (83.33%) than both the even- (20%; χ^2^ (1) = 24.09, *p* < .0005) and better-performance condition (16.67%; χ^2^ (1) = 26.67, *p* < .0005).

For the 20:80 offer, the accept rate was higher in the worse-performance condition (66.67%) than both the better- (13.33%; χ^2^ (1) = 17.78, *p* < .0005) and the even-performance condition (13.33%; χ^2^ (1) = 17.78, *p* < .0005).

For the 10:90 offer, the accept rate was not different between conditions (*ps* > .05).
